# Targeting of β-Arrestin2 to the Centrosome and Primary Cilium: Role in Cell Proliferation Control

**DOI:** 10.1371/journal.pone.0003728

**Published:** 2008-11-14

**Authors:** Anahi Molla-Herman, Cedric Boularan, Rania Ghossoub, Mark G. H. Scott, Anne Burtey, Marion Zarka, Sophie Saunier, Jean-Paul Concordet, Stefano Marullo, Alexandre Benmerah

**Affiliations:** 1 Institut Cochin, CNRS UMR 8104, Université Paris Descartes, Paris, France; 2 INSERM, U567, Paris, France; 3 INSERM, U574, Hôpital Necker-Enfants Malades, Paris, France; 4 Université Paris Descartes, Paris, France; University of Birmingham, United Kingdom

## Abstract

**Background:**

The primary cilium is a sensory organelle generated from the centrosome in quiescent cells and found at the surface of most cell types, from where it controls important physiological processes. Specific sets of membrane proteins involved in sensing the extracellular milieu are concentrated within cilia, including G protein coupled receptors (GPCRs). Most GPCRs are regulated by β-arrestins, βarr1 and βarr2, which control both their signalling and endocytosis, suggesting that βarrs may also function at primary cilium.

**Methodology/Principal Findings:**

In cycling cells, βarr2 was observed at the centrosome, at the proximal region of the centrioles, in a microtubule independent manner. However, βarr2 did not appear to be involved in classical centrosome-associated functions. In quiescent cells, both *in vitro* and *in vivo*, βarr2 was found at the basal body and axoneme of primary cilia. Interestingly, βarr2 was found to interact and colocalize with 14-3-3 proteins and Kif3A, two proteins known to be involved in ciliogenesis and intraciliary transport. In addition, as suggested for other centrosome or cilia-associated proteins, βarrs appear to control cell cycle progression. Indeed, cells lacking βarr2 were unable to properly respond to serum starvation and formed less primary cilia in these conditions.

**Conclusions/Significance:**

Our results show that βarr2 is localized to the centrosome in cycling cells and to the primary cilium in quiescent cells, a feature shared with other proteins known to be involved in ciliogenesis or primary cilium function. Within cilia, βarr2 may participate in the signaling of cilia-associated GPCRs and, therefore, in the sensory functions of this cell “antenna”.

## Introduction

An increasing number of reports have highlighted the function of the primary cilium (PC) in the control of several physiological processes. The PC is a hair-like cellular extension found at the surface of most vertebrate cells. This sophisticated microtubule-based organelle has been shown to sense multiple mechanical and chemical stimuli from the environment and to elicit specific cellular responses, which play crucial roles in embryonic development and homeostatic processes in adulthood. The PC has also recently been implicated in the regulation of cell cycle progression and, as a consequence, a lack of PC was associated with increased proliferation [1]–[3].

PC formation (ciliogenesis) takes place in quiescent or differentiated cells. PCs are assembled from the mother centriole of the unique centrosome present in these cells, which therefore corresponds to the basal body of PC. The mother centriole is docked at the membrane through its distal appendages and gives rise to the microtubule-based 9+0 axoneme, which forms the skeleton of this “antenna” like extension of the plasma membrane. Whereas the basal body shares many properties with classical centrosomes, made of two centrioles and of a pericentriolar matrix, the axoneme represents a unique domain, characterized by the exclusion of many proteins and the enrichment of specific soluble, cytoplasmic, as well as membrane-associated components [1]–[3]. This sorting is achieved through a complex process mediated by highly conserved machineries, involved in both the selection of ciliary proteins, which likely contain specific motifs and the transport along the axonemal microtubule doublets. The components of these machineries can either be specifically devoted to ciliary-protein transport and/or ciliogenesis, as IFT (intraflagellar transport) proteins [4], or participate in other cellular processes, as reported for the aPKC-par3-par6 polarity cassette [5], [6], the 14-3-3 adaptor protein [5] and Kif3A, a kinesin required for the anterograde transport towards the tip of the PC [4]. Polycystins, proteins involved in mechano-sensation of tubular renal cells [7] and growth factor receptors [3] figure among the proteins that are highly enriched in ciliary membranes.

Receptors belonging to the G-protein coupled receptor (GPCR) family are involved in the sensing of many different kinds of molecules including odorants, ions, amines, proteins or light, and thus regulate a large array of physiological processes. Some GPCRs accumulate at the PC, such as the somatostatin type 3 receptor, which is localized at PCs in neurons [8], or smoothened (smo), the GPCR-like transmembrane protein controlling the Hedgehog pathway [9], for which translocation to the PC is essential for signalling activity [10], [11]. Most GPCRs are regulated by non visual arrestins, arrestin2 and arrestin3, also known as β-arrestin1 (βarr1) and β-arrestin2 (βarr2), which uncouple activated receptors from G-proteins, promote their endocytosis through clathrin-coated pits and mediate receptor-dependent activation of MAP kinases [12], [13].

βarrs regulate numerous key physiological and developmental processes as shown by the fact that the lack of both isoforms results in early embryonic lethality [14]. They are highly conserved among higher eukaryotes, although only vertebrates express the two isoforms, which show a high sequence homology and are encoded by two separate genes. βarr isoforms share most of their partners and functions, however, several isoform-specific roles have also been described. In particular, only βarr2 displays an active nucleocytoplasmic shuttling, which redistributes nuclear binding partners to the cytoplasm, whereas regulation of histone acetylation at certain promoters was only reported for βarr1 [15], [16]. Interestingly, βarr2, not βarr1, was found in the cilia of olfactory neurons [17], [18], suggesting that the former might regulate odorant receptors within these structures, which are very similar to PCs. Here, we report that βarr2 is specifically localized to the centrosome of cycling cells. Since most PC-associated proteins are also present at the centrosome in cycling cells, we investigated if βarr2 could also be localized to the PC.

## Results

### βarr2 localizes to the centrosome, in the proximal region of the centrioles, independently of microtubules

**Figure 1:**
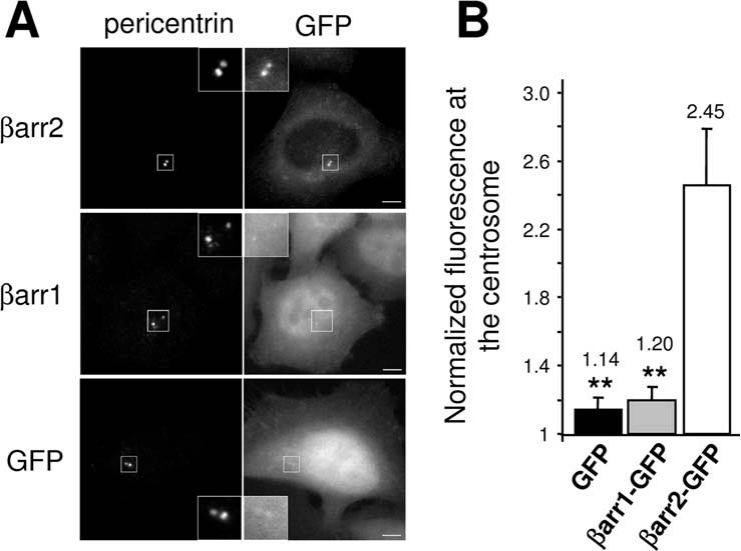
βarr2 is targeted and enriched at the centrosome. (A) HeLa cells were transfected with plasmids encoding for βarr2 or βarr1 GFP fusions or with GFP alone, fixed and stained for the centrosomal marker pericentrin. Insets show higher magnifications of centrosome containing regions. Scale bar represents 5 µm. (B) Centrosome-associated GFP fluorescence was quantified using a region defined by pericentrin staining (see methods) and normalized to the cytoplasmic signal within the same cells. Values are the means (+/− SD) of at least 20 cells from three independent experiments ( ** : p<0.001).

When expressed as a GFP-fusion, βarr2 showed a diffuse cytoplasmic distribution except one or two bright spots close to the nucleus. This localization was suggestive of the centrosome and, confirming this hypothesis, the spots were also decorated with pericentrin (Figures 1A and S1A), a centrosomal marker. The centrosomal targeting of βarr2-GFP was specific, as βarr1-GFP or GFP alone were not enriched at sites of pericentrin staining (Figure 1A and S1A).

The accumulation of βarr2-GFP at the centrosome was quantified, based on GFP-associated fluorescence. Normalized fluorescence at the centrosome for both GFP alone and βarr1-GFP was close to 1 (1.15 and 1.20, respectively), whereas that of βarr2-GFP (2.45) clearly showed accumulation at this organelle (Figure 1B). This new specific localization of βarr2 was independent of the tag fused to the protein, as βarr2 fused with Cherry (βarr2-Ch), a different fluorescent protein [19], was also enriched at the centrosome (Figure S1B).

**Figure 2:**
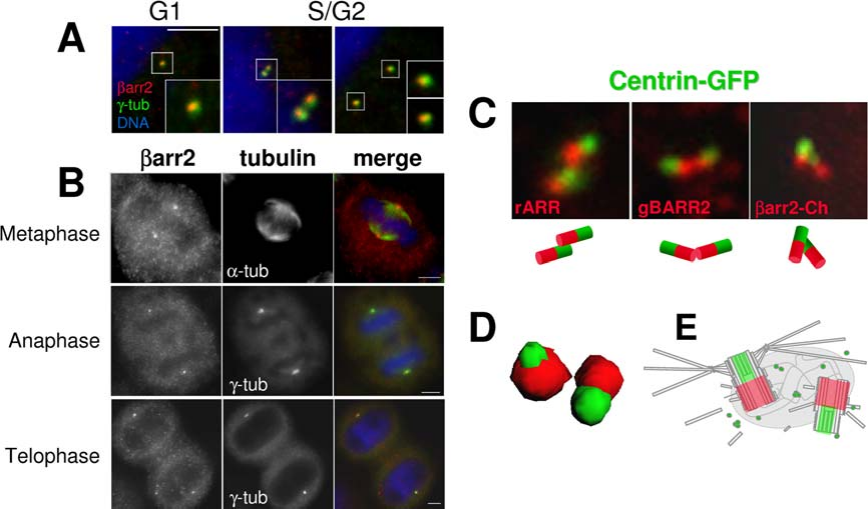
Endogenous βarr2 is localized at the proximal region of centrioles during the overall cell cycle. (A and B) Synchronized HeLa cells were fixed and stained for endogenous βarr2 with the rARR antibody against βarr2 and for γ-tubulin or α-tubulin. DNA was stained with DAPI. Representative images of each step of the cell cycle are shown. In coloured images, βarr2 staining is in red, centrosomes or microtubules in green and nuclei in blue. Insets show higher magnifications of representative areas. Scale bars represent 5 µm. (C) HeLa cells stably expressing a centrin-GFP fusion (green) were either fixed and stained for endogenous βarr2 using either rARR or gBARR2 polyclonal antibodies (red) or transfected with plasmids encoding for the βarr2-Cherry fusion (red), then fixed and directly observed. The possible distribution of each marker was depicted by red and green barrels. (D) Z-stacks images from a representative cell stained with gBARR2 were deconvoluated and a 3D reconstruction is shown. (E) The possible distribution of βarr2 and centrin within the centrosome is illustrated.

Endogenous βarr2 targeting to the centrosome was subsequently investigated using βarr2-specific antibodies and mouse embryonic fibroblasts (MEFs), derived from wild-type (WT), βarr2 (2KO), βarr1 (1KO) and βarr1/2 double knock-out (1/2KO) embryos [14] as controls (Figures S2 and S3). Both antibodies specifically stained bright spots close to the nucleus, which colocalized with pericentrin or γ-tubulin (Figures 2, S3 and S4). In addition, localization of βarr2 at the centrosome was found in both interphasic (Figures 2A and S4) and mitotic cells (Figure 2B). No colocalization was detected with α-tubulin in mitotic cells (Figure 2B), indicating that βarr2 is not associated with the mitotic spindle. Altogether, these results show that βarr2 is associated with the centrosome throughout the entire cell cycle.

The centrosome is composed of two centrioles which are involved in distinct functions [20]. Careful analysis of the staining patterns revealed that the distribution of βarr2 within the centrosome was restricted to structures close to, but distinct from γ-tubulin-containing areas (Figure 2A, insets, and Figure S4B). To more precisely characterize βarr2 localization at the centrosome, we used centrin as a marker of the distal part of centrioles [20]. Both βarr2 antibodies and βarr2-Ch stained two spots juxtaposed to each centrin-decorated structures (Figure 2C), indicating that βarr2 is targeted to the proximal region of centrioles. This specific localization was further confirmed with 3D reconstruction of deconvoluted images, in which juxtaposition of βarr2 and centrin was clearly visible (Figure 2D). Combined, these results establish that βarr2 is targeted to the proximal region of the centrioles (Figure 2E) and that this localization is not modified during the cell cycle.

**Figure 3:**
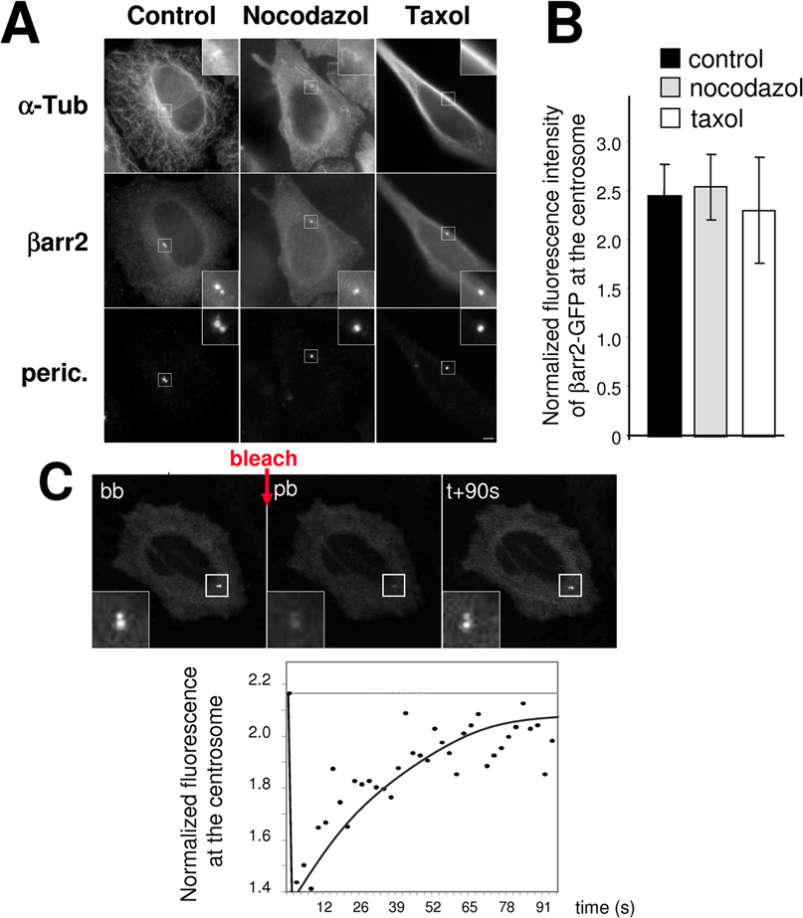
Targeting of βarr2 to the centrosome does not depend on microtubules. (A) HeLa cells expressing βarr2-GFP fusion were untreated (control) or treated with nocodazole or taxol (10 µM) to depolymerize or stabilize microtubule network respectively. Cells were fixed and stained for microtubules (α-tubulin) and for the centrosome (pericentrin). Insets show higher magnifications of regions around centrosomes. Scale bar represents 5 µm. (B) Centrosome-associated fluorescence intensity for GFP was normalized to the cytoplasmic signal in the same cells for each condition. Values are the means (+/− SD) of at least 15 cells from three independent experiments. (C) Live HeLa cells transiently expressing βarr2-GFP fusion were treated for one hour at 37°C with nocodazole (10 µM) then used for FRAP experiments. A small region containing the centrosome was bleached twice with 100% of laser intensity (one second per bleach). Images of a representative cell before (bb), just after bleaching (pb) and 90 seconds after bleaching (+90s) are shown. Insets show higher magnifications of the bleached region. Fluorescence intensity of βarr2-GFP at the centrosome was normalized to cytoplasmic staining within an identical region in the cytoplasm. The FRAP experiments were done in at least 5 cells from two independent transfections.

Targeting of proteins to the centrosome can be achieved through microtubule dependent transport or independently of microtubules by a dynamic exchange with cytoplasm [21]. As shown in Figure 3A, βarr2 was still present at the centrosome in cells treated with nocodazole or taxol, drugs which destabilize or stabilize microtubules, respectively. Normalized fluorescence of βarr2 at the centrosome was indeed similar in drug-treated cells compared to control (Figure 3B), suggesting that targeting of βarr2 to the centrosome is independent of microtubules. This hypothesis was further tested following the dynamic of βarr2 at the centrosome by fluorescence recovery after photobleaching (Figure 3C). Live cells were treated with nocodazole for one hour at 37°C before and during dynamic analysis, a condition which did result in inhibition of microtubule-based transport (Figure S5). In the presence of nocodazole, centrosome-associated βarr2-GFP fluorescence was recovered after photo-bleaching (Figure 3C), with similar kinetics as in control cells (data not shown). These results suggest that the localization of βarr2 at the centrosome likely results from a dynamic exchange between a centrosomal and a cytoplasmic pool.

Since the basic functions of the centrosome are the nucleation and anchoring of microtubules [21], we investigated if βarr2 could affect these processes. Neither process was perturbed by absence or overexpression of βarr2 (Figure S6). In addition, we could not detect any increase of multinucleated cells in βarr-deficient MEFs (data not shown), suggesting that these proteins did not show any role in cytokinesis, another key function of the centrosome [21]. These data therefore suggest that βarrs in general are not involved in classical functions of the centrosome.

### βarr2 is localized to the primary cilium

Since most proteins found at the PC in quiescent cells are found at the centrosome of cycling cells, we investigated whether localization of βarr2 at the centrosome might reflect some role at the PC.

PC formation can be induced *in vitro* in both fibroblasts and RPE1 cells, a widely used model to study ciliogenesis, by growing cells to confluence and this process can be enhanced upon serum starvation (see methods). PC can then be identified using anti-acetylated-tubulin (AT) antibodies, which stain the stabilized array of microtubules forming the axoneme. The basal body, which corresponds to the unique centrosome of these cells, can be identified using centrosomal markers.

**Figure 4:**
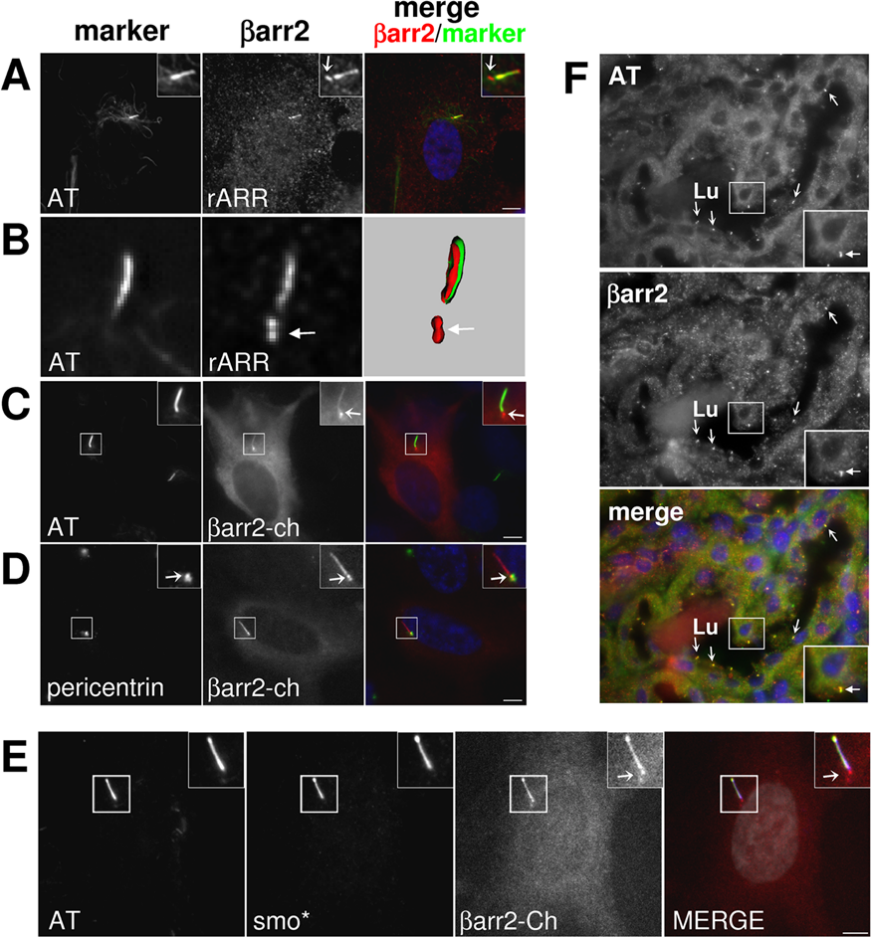
βarr2 is found in the axoneme and basal body of primary cilia. (A) Confluent RPE1 cells were serum-starved for 24 hours, then fixed and stained for acetylated-tubulin (AT), which is highly enriched in primary cilia (axoneme) and for endogenous βarr2 using rARR antibody. (B) Z-stacks images of representative cells were deconvoluated as in Figure 2 and a 3D reconstruction of a representative cilium is shown. (C and D) RPE1 cells transfected with plasmids encoding for the βarr2-Cherry fusion (βarr2-Ch), were serum-starved for 24 hours after transfection, then fixed and stained for AT (C) or for the basal body marker pericentrin (D). In coloured images, βarr2 staining is in red, centrosome or cilia markers in green and nuclei stained with DAPI are in blue. (E) RPE1 cells transfected with plasmids encoding for Flag-tagged active form of smoothened (smo*) and the βarr2-Ch fusion were serum-starved for 24h after transfection, then fixed and stained for AT and smo*, using a rabbit polyclonal anti-Flag antibody. In coloured image, βarr2 staining is in red, AT in blue and smo* in green. Insets show higher magnifications of representative areas. Arrows stress basal bodies. Scale bars represent 5 µm. (F) Tissue sections from adult mouse kidney were stained for AT and for βarr2 using the rARR antibody. In coloured image, βarr2 staining is in red, AT in green and nuclei stained with DAPI in blue. The lumen of a representative tubule is indicated (Lu). Insets show higher magnifications of a representative ciliated tubular epithelial cell. Arrows stress AT positive structures.

After induction of PC assembly in RPE1 cells (Figure 4), endogenous βarr2 was found in the axoneme, as indicated by its colocalization with AT (Figure 4A) and corroborated by 3D-reconstruction of deconvoluated images (Figure 4B). The specific targeting of βarr2 to the axoneme was confirmed using transfected βarr2-Ch which, at low expression levels, did colocalize with AT (Figure 4C). In addition, both endogenous and transfected βarr2 were also present in two spots at the base of the axoneme (Figure 4, arrows), which colocalized with pericentrin (Figure 4D), thus corresponding to the basal body. Finally, βarr2-Ch colocalized with the active form of smoothened (smo*) at the level of the axoneme but not at the basal body (Figure 4E), indicating a possible function in the regulation of cilia-dependent signalling pathways (see discussion).

To establish βarr2 targeting to PC *in vivo*, distribution of βarr2 was analyzed in mouse kidney sections (Figure 4F), where PCs are located at the luminal side (Lu) of tubular epithelial cells [22]. The staining pattern of the βarr2 antibody was similar to that found in cultured cells, showing a colocalization of βarr2 and AT at the apical membrane of epithelial cells (Figure 4F). Together, these data show that βarr2 is targeted to primary cilia *in vivo* and *in vitro*, in both the basal body and the axoneme.

### Lack of βarr2 results in ciliogenesis defects and uncontrolled proliferation

Examples in the literature have established that depletion and/or overexpression of cilia-associated proteins may result in ciliogenesis defects [23]–[26]. We took advantage of the fact that WT MEFs can form PCs to investigate the potential role of βarrs in the control of PC formation.

**Figure 5:**
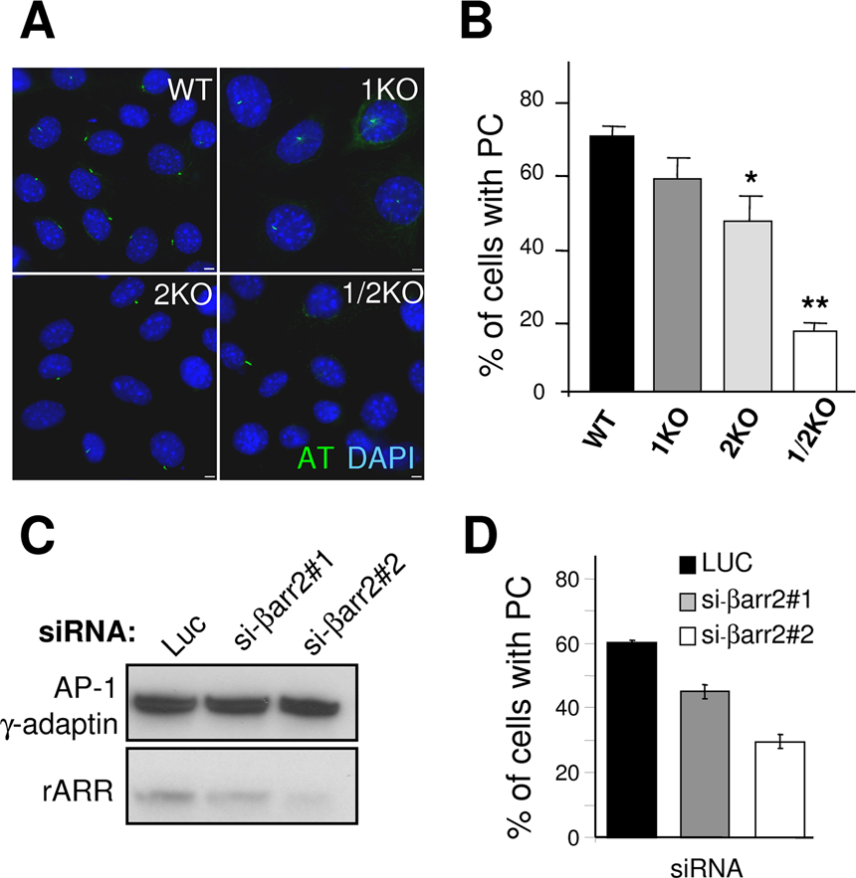
βarr2 deficiency results in ciliogenesis defects. (A) WT, 1KO, 2KO and 1/2KO MEFs were grown in low serum (0,5%) for 48h, then fixed and stained for AT (green). Nuclei were stained with DAPI (blue). (B) The percentage of cells with a normal primary cilium was quantified. Values are the means (+/− SD) of at least 300 cells from three independent experiments (*: p<0,01; **: p<0,001). (C and D) RPE1 cells were treated with control luciferase siRNA (Luc), si-βarr2#1 or si-βarr2#2 to deplete endogenous βarr2. (C) Expression of βarr2 was analyzed by western-blot with the rARR βarr2 antibody. Expression of the γ subunit of the AP-1 clathrin adaptor complex was tested as a control. (D) Cells from the same experiment were also seeded on coverslips and the percentage of cells with primary cilia was determined following AT staining. Values are the means (+/− SD) of ∼300 cells from a representative experiment done in triplicate.

The ability of the 2KO MEFs to form PC upon serum starvation was compared to that of WT MEFs. As shown in Figure 5A and 5B, 76.5% of WT MEFs displayed an assembled PC, indicated by a unique AT-positive 2 µm long rod-like structure, while only 51.5% of the 2KO MEFs exhibited a PC. Similar results were obtained when antibodies against polyglutamylated-tubulin were used to visualize PCs (Figure S7), indicating that the observations based on AT stainings were not just due to side effects of microtubule acetylation.

Because βarrs are redundant for most functions, we investigated the effect of depleting either βarr1 alone or both βarr1 and βarr2 on the number of PCs. MEFs lacking βarr1 were not affected in their ability to form PCs, with a similar proportion of ciliated cells being measured, compared to wild-type cells (Figure 5A and 5B). This result is consistent with the fact that RPE1 cells do not express detectable amounts of βarr1 (Figure S3B) and do form PC (Figures 4, and 5). In contrast, 1/2KO MEFs were greatly impaired in their ability to form PC, with only ∼18% of ciliated cells (Figure 5A and 5B), indicating that ciliogenesis is severely affected in cells completely devoid of βarrs. As expected from these observations, depletion of endogenous βarr2 in RPE1 cells with two different small interfering RNA (siRNA), resulted in markedly reduced ciliogenesis in low serum conditions, compared to a non relevant siRNA (luciferase, Figure 5C and 5D).

A close link likely exists between PC assembly and control of cell cycle progression. It is assumed that only cells exiting the cell cycle and entering into G_0_ phase can form a cilium. On the other hand, ciliogenesis defects result in cell cycle progression and uncontrolled proliferation 2, 27. Since our data indicate that cells lacking βarr2 are affected in their ability to form PCs, we investigated whether these cells would also exhibit defects in proliferation.

**Figure 6:**
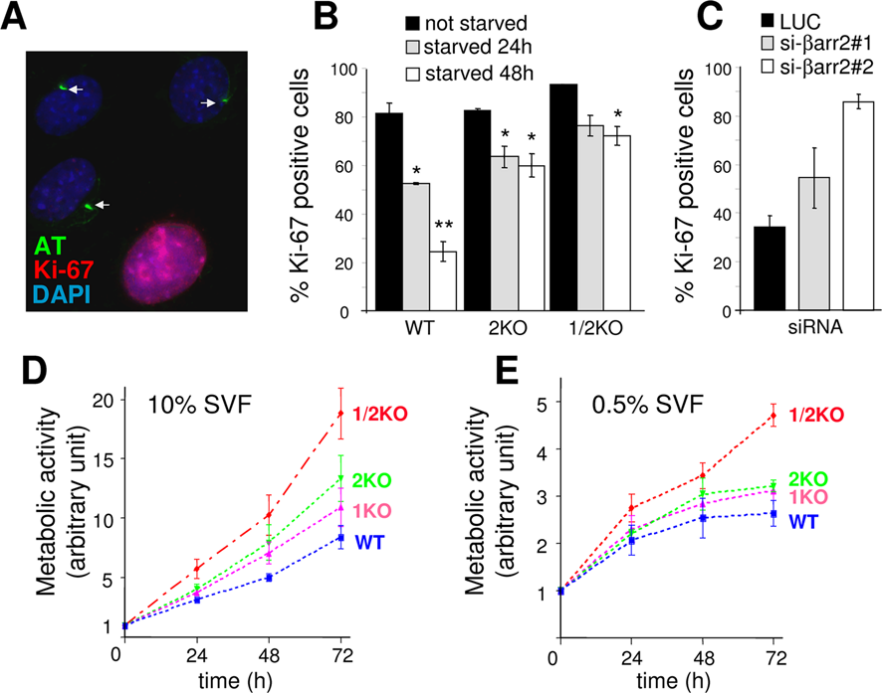
βarr2 deficiency results in uncontrolled proliferation. (A) WT MEFs grown as in Figure 5A were fixed and stained for AT (green) and for Ki-67 (red), a marker of cycling cells (from G1 to M). Arrows stress ciliated cells. (B) WT, 2KO and 1/2KO MEFs were grown in high serum (10%, black bars) or in low serum conditions for 24 (grey bars) or 48h (white bars) and stained for Ki-67. The percentage of Ki-67 positive-cells was quantified for each of the three conditions as indicated in methods. Values are the means (+/− SD) of at least 300 cells from three independent experiments (*: p<0,01; **: p<0,001). (C) RPE1 cells depleted for endogenous βarr2 as in Figure 5 were fixed and stained for Ki-67 and the percentage of Ki-67 positive-cells was quantified as indicated above. Values are the means (+/− SD) of ∼300 cells from a representative experiment done in triplicate. (D and E) WT, 1KO, 2KO and 1/2KO MEFs were seeded at time zero and grown for the indicated time in high (10% SVF, D) or low serum (0,5%, E) conditions. Cell growth was monitored following metabolic activity as detailed in methods.

We first found that the presence of a PC was correlated with exit from the cell cycle and entry into G_0_ phase. Wild-type MEFs (Figure 6A) were grown in low serum for two days after confluence to induce PC formation. Cells were then stained for both AT and Ki-67, a nuclear protein expressed in cells cycling from G_1_ to M [28]. Although in high serum conditions most cells (>80%) showed nuclear Ki-67 staining (Figure 6B), in low serum conditions (Figure 6A and 6B), ciliated cells (arrows) were not positive for Ki-67 (blue nuclei) whereas adjacent non-ciliated cell expressed this proliferation marker (pink (blue and red) nucleus). These results confirmed that ciliated cells were in G_0_ phase.

We subsequently confirmed that ciliogenesis defects observed in βarr2-depleted cells are correlated with cell cycle dysregulation. In WT MEFs, serum starvation induced a decrease of the proportion of cells positive for Ki-67, from 80% in cells grown in high serum, to 50% and 25% for cells grown in low serum for 24 and 48 hours, respectively (Figure 6B). Interestingly, the percentage of Ki-67 positive cells was inversely correlated with the number of ciliated cells (compare with Figure 5B). When 2KO and 1/2KO MEFs were grown in the same conditions, the number of cells positive for Ki-67 moderately decreased upon serum starvation but remained constant from 24 to 48 hours with 60 to 70% of the cells remaining positive for Ki-67 at 48h (Figure 6B). Since the percentage of ciliated cells was decreased in βarr2 deficient cells (Figure 5B), it appears that the defect in PC formation is correlated with an absence of response to low serum conditions and impaired exiting from cell cycle to enter in the G_0_ phase. Similarly, an increased proportion of Ki-67 positive cells was also found in RPE1 cells depleted for βarr2 and grown in low serum conditions (Figure 6C).

In cystic kidney disease, a pathological condition associated with impaired formation and/or function of PCs, cyst formation is due to both loss of planar polarity and increased mitosis in tubular cells [22]. Moreover, recent studies showed that Polaris, a protein responsible for a mouse model of polycystic kidney disease, controls cell cycle progression [26]. When MEFs were grown in high serum conditions, 2KO and 1KO cells showed increased proliferation compared to wild type cells, a phenotype more pronounced in 1/2KO cells (Figure 6D, p<0.01 at 72h). Interestingly, cells lacking both βarr isoforms kept growing even in low serum conditions (Figure 6E, p = 0.001 at 72h). Therefore, the marked defect in ciliogenesis observed in 1/2 KO cells is likely to result from the inability of these cells to respond to signals that inhibit cell proliferation.

### βarr2 interacts with 14-3-3 proteins and kinesin Kif3A

14-3-3 proteins (comprising β, γ, ε, η, ζ, τ and σ isoforms) are molecular adaptors, which often interact with consensus phosphorylated serine/threonine motifs of many proteins, thereby controlling a wide array of processes including signalling, cell cycle and apoptosis [29]. Interestingly, the 14-3-3ζ isoform was found to interact with the aPKC-Par3-Par6 polarity cassette [30], whereas depletion of 14-3-3η, which was found in a molecular complex with Par3 and the kinesin Kif3A, resulted in ciliogenesis defects [5]. Unpublished yeast two-hybrid data revealed that βarrs interact with 14-3-3 proteins, in agreement with a recent proteomic study [31]. The implication of 14-3-3η in ciliogenesis and its connection with intraciliary transport through Kif3A, prompted us to characterize these interactions with βarr2.

**Figure 7:**
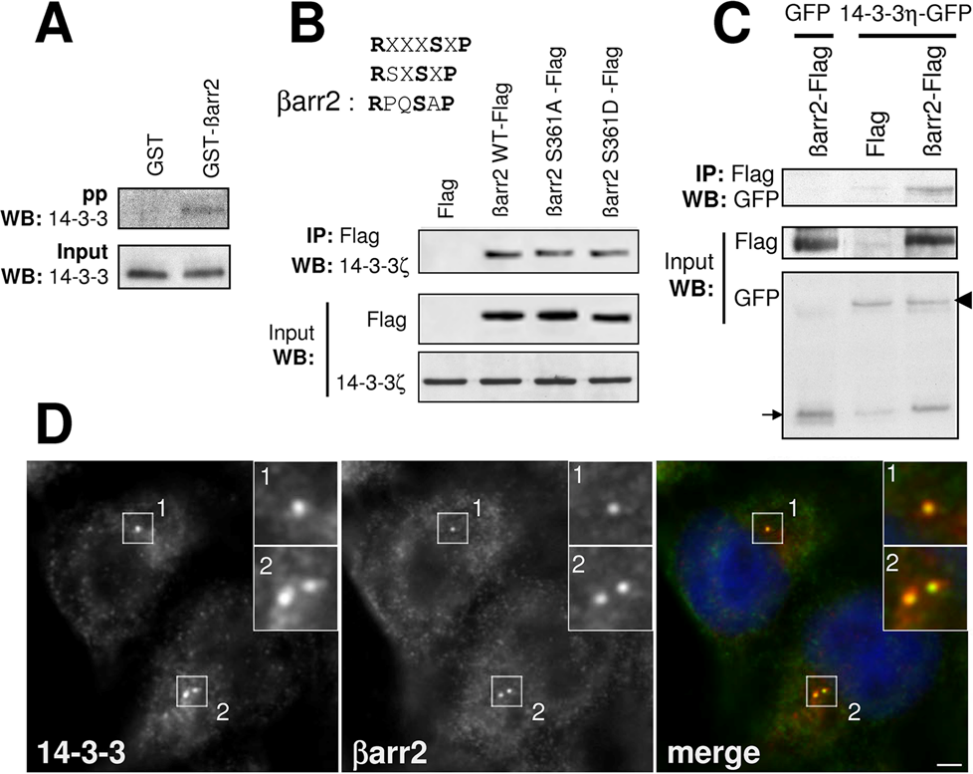
βarr2 interacts with 14-3-3 proteins. (A) Cell lysates were precipitated with GST or GST-βarr2 fusion and the presence of 14-3-3 proteins in the precipitates was revealed by western-blot (WB) with a polyclonal antibody which recognizes all 14-3-3 isoforms. (B) A unique putative phosphorylation dependent 14-3-3 binding motif was identified in βarr2 (RPQSAP). Lysates from cells transfected with either Flag vector, WT βarr2, βarr2-S361A or βarr2-S361D Flag-tagged constructs were immunoprecipitated with anti-Flag antibody, and the precipitated proteins were analysed by western-blot (WB) with an antibody directed against 14-3-3ζ. (C) Cell lysates from cells expressing either GFP and βarr2-Flag or GFP-14-3-3η and Flag or βarr2-Flag were immunoprecipitated with the anti-Flag antibody and immunoprecipitated proteins were analysed with an anti-GFP antibody. Arrow and arrowhead stress GFP and GFP-14-3-3η, respectively. (D) RPE1 cells were fixed and stained for βarr2 (gARR2, green) and 14-3-3 antibody (red). Insets show higher magnifications of representative areas. Scale bar represents 5 µm.

Endogenous 14-3-3 proteins were precipitated by a GST-βarr2 fusion (Figure 7A) and the interaction between βarr2 and the 14-3-3ζ isoform was confirmed by co-immunoprecipitation experiments showing that endogenous 14-3-3ζ interacts with Flag-tagged βarr2 (Figure 7B). The βarr2 C-terminus contains a motif (RPQSAP), similar to phosphorylated 14-3-3 consensus binding sites (Figure 7B). However, mutation of S361 within this motif did not affect the interaction of βarr2 with endogenous 14-3-3ζ, which co-immunoprecipitated as efficiently with both the S361A and S361D mutants of βarr2 (Figure 7B), indicating that the interaction of βarr2 with 14-3-3 might be constitutive. This hypothesis is consistent with the observations that 14-3-3 proteins interact with recombinant GST-βarr2 and that the 14-3-3/βarrs interaction was not affected by GPCR activation (data not shown and ref [31]). A phosphorylation-independent interaction of βarr2 with 14-3-3 proteins would not be unique, since it has already been reported for other partners of 14-3-3 [29].

Interaction of βarr2 with 14-3-3ζ was extended to the other isoforms and we found that flag-tagged βarr2, could co-immunoprecipitate with almost all 14-3-3 proteins (data not shown), including 14-3-3η (Figure 7C), the isoform which has been implicated in ciliogenesis [5]. Finally, the possible colocalization of 14-3-3 proteins with βarr2 was analyzed at the centrosome and PCs. In contrast to what was observed in kidney cells [5], 14-3-3 proteins were not detected on the axoneme of PCs in RPE1 or MEFs. In these cells, 14-3-3 proteins were only found at the centrosome or basal body where they colocalized with γ-tubulin (Figures S8 and data not shown) and with βarr2 (Figure 7D and data not shown).

**Figure 8:**
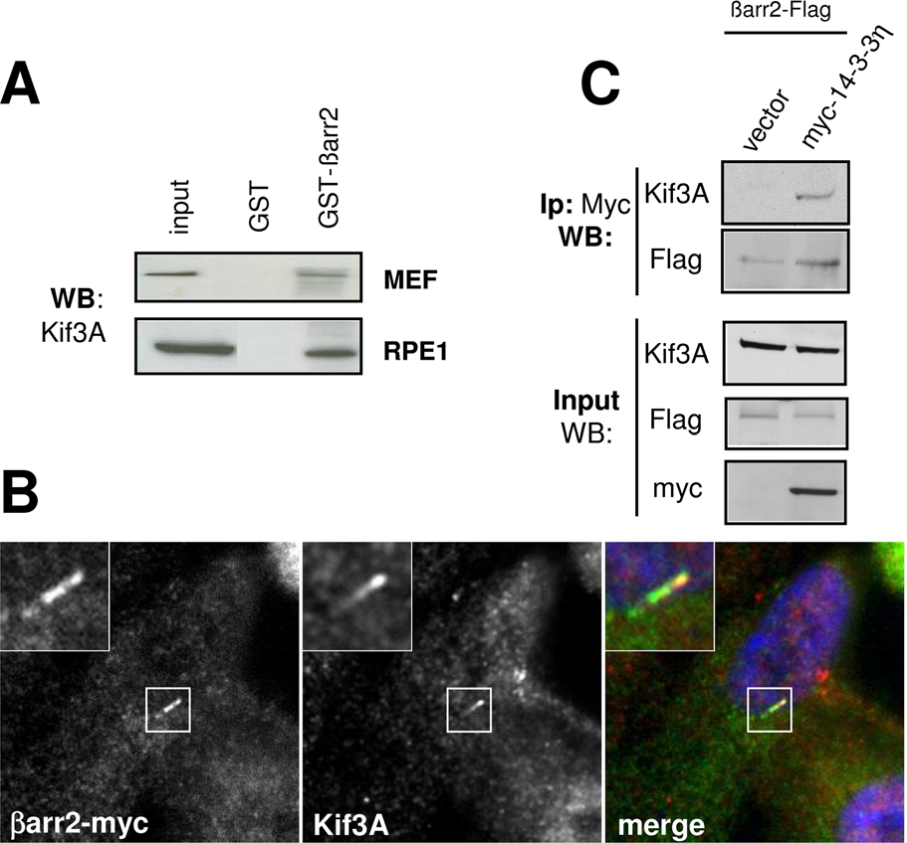
βarr2 interacts with Kif3A. (A) RPE1 or MEFs cell lysates were precipitated with GST or GST-βarr2 fusion and the presence of Kif3A in the precipitates was revealed by western-blot (WB). (B) RPE1 cells grown on coverslips were transfected with βarr2-myc, grown in low serum and then fixed and stained for Kif3A (red) and anti-Myc (green). Insets show higher magnifications of a representative PC. (C) COS cells transfected with either βarr2-Flag construct or vector alone together with myc-tagged 14-3-3η were lysed and cell lysates were immunoprecipitated with the anti-Myc antibody. The precipitated proteins were analysed by western-blot (WB) with antibodies against Flag, Myc or Kif3A.

Endogenous Kif3A was also precipitated by GST-βarr2 (Figure 8A), an interaction confirmed by colocalization studies. Indeed, as reported *in vivo* [32], Kif3A was found in the cytoplasm and at the tip of the axoneme where it was colocalized with βarr2 (Figure 8B). Finally, because Kif3A was reported to co-immunoprecipitate with 14-3-3η [5], [30], we investigated whether βarr2 could be present in the same molecular complex. Supporting this hypothesis myc-14-3-3η co-immunoprecipitated with both Flag-tagged βarr2 and endogenous Kif3A (Figure 8C).

## Discussion

Our data show that βarr2 shares many of the hallmarks of proteins found at the primary cilium or involved in ciliogenesis: it is targeted to the centrosome in cycling cells and to the basal body and axoneme of PC in quiescent cells; its depletion results in accelerated and uncontrolled cell growth resulting in impaired ciliogenesis.

Similar to many PC proteins, such as, Polaris/IFT88, IFT20 or IFT57 [26], [33], βarr2 was found at the centrosome in cycling cells and more precisely at the proximal part of the centrioles (Figures 1 and 2). Although we could not find evidence for a role of βarr2 in the basic functions of the centrosome, βarr2 shares with other centrosome-associated proteins a role in cell cycle regulation. The centrosome participates in several different cell cycle regulatory events, such as G1/S transition, cytokinesis, and monitoring of DNA damage, functions which involve the recruitment of specific sets of proteins [34]. Recent studies showed that depletion of structural centrosomal proteins, such as PCM-1 or pericentrin results in a p53-dependent G1/S arrest [35], [36], suggesting that the centrosome itself is involved in cell cycle control. Consistent with these observations, we found that βarrs-deficient cells do not respond properly to serum starvation, as shown by their persistent growth in low serum and their failure to enter in G_0_ phase, whereas they proliferate faster in high serum conditions (Figure 6). The strong additive effect of the simultaneous depletion of both βarrs likely reflects the fact that each isoform may have specific points of impact.

A role of βarr1 was reported in G1/S transition downstream of IGF receptor [37], and via a receptor-independent enhancement of p27 transcription, which, in turn, inhibits G1/S transition [38]. Consistent with our observations on 1KO MEFs (Figures 5 and 6), depletion of βarr1 in the latter study was shown to increase cell proliferation. βarr2 was also reported to control cell growth in response to nerve growth factor in PC12 cells [39]. However, the mechanism by which βarr2 controls cell cycle appears to be different. βarr2 interacts with mdm2, the E3 ubiquitin ligase controlling the stability of p53, a transcriptional factor which plays a major role in cell cycle regulation [40]. βarr2 was specifically reported to actively exclude mdm2 from the nucleus [41], to stabilize p53 by this mechanism, leading to either induction of apoptosis [41] or cell cycle arrest at G2/M transition [41], [42]. Thus, the lack of βarr2 would favour destabilization of p53 and then promote cell cycle progression and increased proliferation, as observed here in cells depleted for βarr2 (Figures 5 and 6). Altogether, these data support the idea that βarrs have distinct but converging roles in cell cycle regulation and control of cell proliferation.

Our results strikingly paralleled those reported on Polaris, a protein found at the centrosome in cycling cells, which is involved in intra-ciliary transport and required for ciliogenesis. Overexpression of Polaris prevented G1/S transition and induced apoptosis, whereas its depletion promoted cell-cycle progression and increased cell growth [26]. A role in cell cycle control is shared by other IFT proteins such as IFT27 [43] but not all [33]. Altogether, these notions highlight the functional connections between centrosome-associated proteins, IFT proteins and p53 and highlight the centrosome as a meeting point for both proliferative and anti-proliferative controllers [34].

Another similarity between βarr2 and Polaris is that the increased proliferation observed in βarrs-depleted cells is correlated with reduced ciliogenesis in response to serum starvation, as observed in βarr-deficient MEF and siRNA-treated RPE1 cells (Figures 5 and 6 and ref [26]). In a recent study, which also reported the localization of βarrs at PC, βarrs-deficient MEF cells did not show ciliogenesis defects [44]. The discrepancy with our findings is likely due to differences in cell culture conditions. Indeed, the authors of the previous study found that only 20% wild-type MEF cells formed PC, consistent with what was previously described for MEF grown in high serum conditions [45]. In the present study, we analyzed ciliogenesis in response to serum starvation, a widely used experimental condition to induce PC assembly. In these conditions we repeatedly observed that ∼70% of wild-type MEF cells were ciliated (Figure 5), as described elsewhere [46]. Altogether, these data suggest that the effect of βarrs expression on ciliogenesis is only observed upon serum starvation.

In addition, from our data, it appears that the effect on ciliogenesis is likely linked to uncontrolled proliferation rather than direct effect on the ciliogenesis itself. We observed a striking correlation between the inability to enter in G_0_ in response to serum starvation, increased proliferation and reduced ciliogenesis. Therefore, if βarr-deficient cells are unable to enter in G_0_ even in response to serum starvation they would not be able to build a cilium. In addition, we observed an increased proportion of cycling cells among ciliated single βarr1 and βarr2 KO cells (Figure S9). Interestingly, an aberrant outgrowth of PC in cycling cells was recently described upon depletion of Cep97 and CP110 proteins [47]. These data suggest that depletion of βarrs may also result in a disconnection between the presence of a PC and cell cycle arrest.

Although the βarr-dependent control of PC formation reflects the role of these proteins on cell proliferation, a direct role in ciliogenesis or in the transport of PC proteins cannot be excluded. The constitutive interaction of βarr2 with 14-3-3 and Kif3A (Figures 7 and 8), which are both involved in ciliogenesis, can support this hypothesis. In addition, βarr2 interaction with Kif3A and 14-3-3 proteins might control its transport within PCs. Indeed, in photoreceptor cells, visual arrestin (varr) regulates the signalling activity of rhodopsin, the light-sensing GPCR. In response to light varr is transported from the inner segment (cell body) to the outer segment (rhodopsin containing compartment), through the connecting cilium, which is a modified PC [48]. In the absence of Kif3A, varr is unable to reach the outer segment in response to light [49], suggesting that Kif3A is responsible for its transport through the connecting cilium. Finally, in the absence of light, varr is also localized in the connecting cilium and at the basal body [50]–[53], paralleling our observations showing a constitutive targeting of βarr2 to PC. The possible role of Kif3A in the targeting of βarr2 to PC could not be tested in our cellular models, since depletion of Kif3A resulted in major ciliogenesis defects (Figure S10) as previously reported [25], [32], [54].

While this article was in preparation, a recent study reported that βarrs mediate the interaction of smo with Kif3A leading to the targeting of active smo to PC [55]. Whether this observation could account for a more general function of βarrs in the targeting of GPCRs to PCs is a key question to be addressed. Our preliminary results indicate that the Somatostatin type 3 receptor (SST3R), a GPCR described in neuronal PC [8], was efficiently targeted to the axoneme in βarr-deficient cells (Figure S11). These data suggest that βarrs are not implicated in the targeting of all GPCRs to PC. Interestingly, contrasting with what was reported for smo, activation of SST3R is not required for PC targeting, which rather appears constitutive (Figure S11 and [56]) and dependent on Bardet Bield Syndrome proteins [56].

Another open issue is the exact function of βarr2 within the cilium in the context of GPCRs physiology. Our data indicate that βarr2 is constitutively localized to PC: it was found within the axoneme in serum-starved cells and this localization was not modified by the expression of constitutively active form of smo (Figure 4 and data not shown). One of the key functions of βarr2 at the plasma membrane is to mediate internalization of agonist-activated GPCRs through clathrin-coated pits. However, despite the presence of clathrin-coated pits at the base of primary cilia (unpublished observations), there is no evidence in the literature for internalization of proteins found in the membrane of PC. Finally, βarr2 might participate in the desensitization of activated GPCRs localized at PC, as shown for odorant receptors in olfactory neurons [17], [57], [58] and reminiscent of the function of varr in the outer segment of visual cells. Interestingly, activated rhodopsin of the outer segment is not internalized in response to light [48], suggesting that in PCs, receptors might be sequestered from classical downregulation pathways involving clathrin-mediated endocytosis.

## Materials and Methods

### Plasmids

Constructs encoding for GFP fusions of wild-type βarr2 (βarr2-GFP) and βarr1 (βarr1-GFP), Cherry-tagged (βarr2-Ch), GST fusion as well as Flag- and Myc-tagged βarr2 were described previously [42], [59]. Point mutant S361A or S361D within rat βarr2-Flag fusion was generated as described previously [42], [59]. Rat wild-type smoothened (smo) cloned into pcDNA3 (Invitrogen) was provided by Dr J Coulombe (Institut de Neurobiologie, Gif-sur-Yvette, France). Flag-tagged constitutively active form of smo (smo*) was generated according to [60]. GFP-tagged 14-3-3η was provided by Drs C Brock and JP Pin (Institut de Génomique fonctionnelle, Montpellier, France) and further subcloned into pCMV-Tag3A (Stratagene, La Jolla, CA, USA) to generate myc-14-3-3η. Flag-tagged form of the somatostatin type 3 receptor (SST3R) was a kind gift of Dr. W. Meyerhof (German Institute of Human Nutrition, Potsdam-Rehbrücke, Germany).

### Cells

HeLa cell line stably expressing Centrin-GFP [61] and RPE1, a human retinal pigment epithelial cell line that stably expresses human telomerase reverse transcriptase (hTERT-RPE1; CLONTECH Laboratories, Inc.) were gifts from M. Bornens (Institut Curie, Paris, France). Mouse embryonic fibroblasts from wild-type or from βarr2 or βarr1, or βarr2 and βarr1 knock out mice [14] were a kind gift of RJ Lefkowitz (Howard Hughes Medical Institute, Durham, USA). HeLa and COS-1 cell lines were from ATCC. Cells were grown in Dulbecco's Modified Eagle's Medium (DMEM) supplemented with 10% fetal bovine serum (Invitrogen) except RPE1 which were grown in DMEM-F12 1:1 supplemented by 10% fetal bovine serum (Invitrogen).

### Antibodies

Mouse monoclonal antibody against polyglutamylated-tubulin (GT335, [62]) was a kind gift of D. Boucher (Université Pierre et Marie Curie, Paris, France). Rabbit polyclonal antibody against Pericentrin (ab4448), 14-3-3 isoforms (ab9063), goat polyclonal antibody against βarr2 (ab31294), and 9E10 anti-Myc mouse monoclonal (ab32) were from Abcam. Arrestin rabbit polyclonal antibody was from Abcam (ab2914) or ABR-affinity-bioreagents (PA1-730). Mouse monoclonal antibodies against γ-tubulin (clone GTU-88), α-Tubulin (clone DM1A), acetylated-tubulin (clone 6-11B-1), γ-adaptin subunit of the AP-1 clathrin adaptor complex (clone 100.3); rabbit polyclonal antibody against Kif3A; as well as mouse monoclonal and rabbit polyclonal antibodies against the Flag epitope were from Sigma. Rabbit polyclonal antibody against Ki-67 was from Novocastra (NLC-Ki-67p, Menarini Diagnostics, Novocastra Laboratories ltd, United Kingdom). Rabbit polyclonal antibodies against 14-3-3ζ (sc-1019) and α-adaptin of the AP-2 clathrin adaptor complex (sc-10761) were from Santa Cruz Biotechnologies. Mouse monoclonal antibodies against βarr1 (clone 10, ref 610551) was from BD-biosciences. Mouse monoclonal anti-GFP antibody was from Jackson ImmunoResearch.

Alexa Fluor conjugated secondary antibodies were from Molecular Probes (Invitrogen). Cy3-labeled donkey anti-goat antibody and horseradish peroxidase–conjugated donkey anti-rabbit or anti-mouse IgG were from Jackson ImmunoResearch.

### Microtubules and induction of primary cilia

HeLa cells were synchronised by a treatment with nocodazole (1 µM) overnight and further release by transfer into basic culture media. Microtubule re-growth experiments were performed on MEFs and transfected HeLa cells. Briefly, cells grown on coverslips were treated with nocodazole (10 µM) for 45 minutes at 4°C or with Taxol (10 µM) for 45 minutes at 37°C to depolymerize or stabilize microtubules respectively. Treated cells were then either immediately fixed after a rapid wash in cold PBS, or washed twice in warmed PBS, then incubated for 5 or 10 minutes in pre-warmed (37°C) serum free DMEM, and finally fixed. Nocodazole and Taxol were from Sigma.

To induce ciliogenesis, MEFs or RPE1 cells were grown to confluence on coverslips treated (for MEFs) or not with polylysine (Sigma) in the presence of serum and then grown in low serum containing media (0,5% FBS) for 24 or 48 hours.

### Transfections

Transfections were done following the recommended procedure of the Genejuice (Novagen) or of the FuGENE HD (Roche) transfection reagents. Basic transfection conditions were used for HeLa and HeLa-Centrin-GFP. MEFs or RPE1 cells were grown on coverslips up to 70%, then transfected and immediately grown in low serum conditions.

For siRNA experiments, RPE1 cells were treated with previously described control siRNA (Luciferase, Luc) or which target βarr2 (si-βarr2#1) or both βarr1 and βarr2 (si-βarr2#2) oligos [63], [64] using a protocol described elsewhere [65]. For the targeting of Kif3A, a smart pool from Dharmacon (ON-target plus SMART pool L-004964-00-0005) was transfected following the same protocol. Briefly, siRNA duplexes were transfected using Oligofectamine (Invitrogen) according to the manufacturer's instructions. Sub-confluent RPE1 cells (70%) were transfected the first day with 200 pmol of siRNA, then splitted the day after and transfected again with 200pmol of siRNA. Transfected cells were grown in low serum (0.5%) the third day and then processed for immunofluorescence or biochemistry on the fourth day.

### FRAP analysis

Dynamic of βarr2 at the centrosome was analyzed by FRAP (fluorescence recovery after photobleaching). Hela cells expressing βarr2-GFP were treated or not with nocodazole (1h, 37°C, 10 µM) and analyzed using a laser scanning confocal microscope (TCS SP2 AOBS, Leica) after excitation with a 488-nm laser line from an argon laser as previously described [66]. Briefly, a region containing the centrosome was exposed to two consecutive pulses of 1 second with 100% of the laser intensity, and recovery of fluorescence was analyzed for 90 seconds taking an image every 1.3 seconds. Images were then analyzed using Metamorph to quantify normalized fluorescence at the centrosome (see Methods). The final images were generated using NIH image (http://rsb.info.nih.gov/nih-image/) or scion image (http://www.scioncorp.com) and Photoshop (Adobe Systems Inc.).

### Immunofluorescence and immunohistochemistry

Cells grown on coverslips were washed twice in PBS and fixed in methanol (methanol/acetone: 1/1) at −20°C for 5 minutes or in 4% paraformaldehyde (PFA) for 30 minutes at 4°C followed by a 10 minutes incubation in PBS-NH_4_Cl (50 mM). Cells were incubated with primary antibodies in permeabilization buffer (PBS with 1 mg/mL bovine serum albumin (PBS-BSA) and 0.1% triton-X-100 (Sigma)) for 45 minutes at room temperature. After two washes with PBS-BSA, cells were incubated for 30 minutes at room temperature in PBS-BSA containing secondary antibodies. After one wash with PBS-BSA and two washes in PBS, cells were laid down on microscope slides in a PBS–glycerol mix (50/50) using the SlowFade Light Antifade Kit with DAPI from Molecular Probes (Invitrogen).

Kidneys from 4 weeks old-mice were harvested and embedded in OCT and snap-frozen in isopentane/liquid nitrogen for cryostat sections. Immunofluorescence labelling was performed on 6-µm-thick sections fixed in acetone for 10 minutes, and incubated over night at 4°C with anti-βarr2 or anti-AT antibodies diluted in incubation buffer (PBS-BSA; 0.1% triton containing 10% donkey serum). A mounting media containing DAPI (VECTASHIELD, Vector Laboratories, Burlingame, CA) was used to label the nuclei.

Samples were examined under an epifluorescence microscope (Leica, Reuil Malmaison, France) with a cooled charge-coupled device (CCD) camera (Micromax, Roper Scientific, Evry, France). Images were acquired with MetaMorph (Universal Imaging, Downingtown, PA, USA) and processed with MetaMorph and Photoshop (Adobe Systems Inc., San Jose, CA, USA).

### Image analysis using Metamorph

To calculate normalized fluorescence of GFP or Cherry fusions at the centrosome, transfected HeLa cells were fixed and stained for pericentrin and the pericentrin staining was then used to define a region corresponding to the centrosome. Briefly, the option “Auto Threshold for light objects” allowed us to transform stainings in objects which were then circled by selecting the option “Create regions around objects” in Metamorph. The resulting regions were then transferred to the GFP or Cherry corresponding images, and the fluorescence intensity corresponding to GFP/Cherry in the centrosome (CE) was measured. To normalize these values to the local background, a region of the same size was selected outside the cell to measure the noise of the camera (CN) and another one in the cytoplasm (CY) of the same cell allowing us to normalize centrosome-associated signal to the expression level of the GFP/Cherry fusions in each cell. Normalized fluorescence at the centrosome was then calculated as follows: NF = (CE−CN)/(CY−CN). To measure the expression level of Ki-67 in nuclei of MEFs, DAPI staining was used to define a region corresponding to the nucleus of each cell as indicated above for centrosomes. Ki-67-associated fluorescence was then measured within these regions. To discriminate between Ki-67 negative and positive cells, the average fluorescence intensity of Ki-67 was measured in ciliated cells and nuclei were considered as positive if their Ki-67-associated fluorescence was above this average value.

### Deconvolution

Epifluorescence images were obtained with an epifluorescence microscope (Zeiss) using a 100× objective (plan-apo) coupled to a “piezzo” enabling acquisition of images every 200 nm in the Z plane. Deconvolution of z-stacks was achieved with metamorph and 3D reconstruction of deconvoluated images with the Imaris software (Bitplane, Scientific solutions, Zurich, Switzerland and Minneapolis, USA). Movies or single images can be extracted from Imaris and then used to obtain the final views used in the figure.

### Proliferation Test

The proliferation test was performed using UptiBlue reagent (Interchim, Montluçon, France) according to the manufacturer's instructions. Briefly, MEFs cells were seeded at 1000 cells/well (100 µL) in 96-well microtiterplates. Each assay was performed in triplicate. After 0, 24, 48 or 72 hours, 10 µL of UptiBlue working solution was added to each well and fluorescence was read at 590 nm on Typhoon© 9400 scanner (GE Healthcare, Piscataway, NJ, USA; with settings: excitation laser at 532 nm, filter 580BP30, PMT 350 V). For each cell line, proliferation rate was determined as a ratio of the fluorescence intensity emitted at λ = 590 nm for time t less associated background above the fluorescence intensity emitted for time t = 0 less associated background.

### Immunoprecipitation and immunoblotting

For Western Blot experiments, cells were lysed by incubation in lysis buffer (0.02M Tris HCl pH 7.5, 1% NP40, 0,1 M NH_4_SO_4_, 10% Glycerol, 10 mM protease inhibitor cocktail (Sigma)) for 30 minutes at 4°C. After centrifugation, cleared lysates were separated by polyacrylamide gel electrophoresis (SDS-PAGE) and transferred onto polyvinylidene fluoride transfer membranes (PVDF, GE Healthcare) using the NuPage electrophoresis system (Invitrogen). Immunoblotting was performed using the indicated primary antibodies and revealed using the ECL^+^ Detection Kit (GE Healthcare).

For immuno-precipitation, COS-1 cells transiently transfected with the indicated constructs were lysed as indicated above and cell lysates (500 µg of proteins) were incubated at 4°C for 12h with 20 µL of the monoclonal M2 anti-Flag affinity agarose (Sigma) or 1 µg of 9E10 anti-myc antibody. The immunoprecipitates were then washed twice with buffer 1 (1% NP-40, 0.5% deoxycholic acid, 50 mM Tris (pH 7.5), 150 mM NaCl), twice with buffer 2 (0.1% NP-40, 0.05% deoxycholic acid, 50 mM Tris (pH 7.5), 500 mM NaCl), and twice with buffer 3 (50 mM Tris (pH 7.5), 0.1% NP-40, 0.05% deoxycholic acid), and analyzed by Western-blot as explained above.

GST-βarr2 fusion protein and GST were expressed in BL21(DE3)pLysS (Invitrogen) and purified on a GSTrap FF column (GE Healthcare) according to the manufacturer's instructions. GST fusions were eluted with 10 mM glutathione were desalted on a HiTrap desalting column (GE Healthcare) in PBS and analyzed by 10% SDS-PAGE and Coomassie blue staining. For *in vitro* binding assays, 25 µg of GST fusion proteins were immobilized on 20 µl glutathione-Sepharose beads for 1h at 4°C in PBS. Beads were washed twice in 1 ml PBS and twice in binding buffer (50mM Tris HCl pH7.5, 150 mM NaCl, 1% NP40, 0.5% sodium deoxycholate supplemented with protease inhibitors (Sigma)). Cell lysates (500 µg of total proteins) were then incubated for 12 h at 4°C with GST loaded beads, then washed twice with buffer 1, twice with buffer 2, and twice with buffer 3. Complexes were separated on 10% SDS-PAGE, transferred proteins were revealed by Ponceau Red staining of the membrane and precipitated proteins were analyzed by Western blot.

## Supporting Information

Figure S1 Targeting of GFP and Cherry βarr2 fusion at the centrosome in RPE1 cells. RPE1 (retinal pigment epithelial) cells were transiently transfected with plasmids encoding for βarr2-GFP fusion or GFP alone (A), or βarr2-Cherry fusion or Cherry alone (B), then fixed and stained for the centrosomal marker pericentrin. Insets show higher magnifications of representative areas. Scale bar represents 5µm. (C) Pericentrin staining was used to determine the centrosome-associated fluorescence intensity for GFP or Cherry (see Methods) which was then normalized to the cytoplasmic signal in the same cells. Values are the means (+/− SD) of at least 15 cells from three independent experiments ( **: p<0.001).
(8.56 MB TIF)

Figure S2 Characterization of anti-βarr2 antibodies. Description of the immunogenic peptides used to generate anti-βarr2 polyclonal antibodies: The rARR rabbit polyclonal antibody is sold as an antibody against both βarr2 and βarr1 but a single amino-acid difference in the immunogenic peptide makes it more specific for βarr2. The gBARR2 goat polyclonal was raised against a peptide specific of human βarr2 and not conserved in βarr1. However, a single amino acid difference between human and rodent βarr2 is likely to explain its poor reactivity against murine endogenous βarr2 observed in both western blot and immunofluorescence (data not shown). HeLa cells were transiently transfected with plasmids encoding for βarr2-GFP, then fixed and stained for the anti-βarr2 antibodies, including the rabbit polyclonal rARR anti-arrestin (A) and the goat polyclonal gBARR2 anti βarrestin2 (B). In coloured images, βarr2-GFP staining is in green, endogenous βarr2 in red and nuclei stained with DAPI are in blue. Insets show higher magnifications of representative areas corresponding to the centrosome containing region of cells expressing (1) or not (2) GFP-βarr2 in the same field. GFP-βarr2 expressing cells showed increase staining with the anti-βarr2 antibodies showing that they did work for immunofluorescence. In non-transfected cells, the antibodies showed a diffuse staining in the cytoplasm and illuminated two bright spots, suggesting that both antibodies are able to detect both overexpressed and endogenous βarr2. Scale bars represent 5µm.
(9.82 MB TIF)

Figure S3 The rARR antibody is specific for βarr2 and stains the centrosome. It has to be stressed here that, independently of the commercial source, we observed a variability between batches of commercial anti-βarr2 antibodies; while almost all batches did detect overexpressed βarr2, some were unable to detect endogenous βarr2 in neither western-blot or immunofluorescence experiments. The efficiency of each batch was then tested by western-blot using WT and βarrs-KO MEFs as described below. (A and B) βarr2 expression was assessed in mouse embryonic fibroblasts (MEFs) derived from wild type (WT), βarr2 deficient (2KO), both βarr1 and βarr2 deficient (1/2KO) mice, RPE1 (retinal pigment epithelial cells) or HeLa cells by western blotting (WB) using the rabbit polyclonal antibody against βarr2 (rARR, (A)) or a monoclonal antibody against βarr1 (mβ1, (B)). An antibody against α-adaptin subunit of the clathrin adaptor complex AP2 was used as a loading control. (C) WT or 1/2KO MEFs were fixed and stained for the centrosomal marker γ-tubulin (γ-tub) and endogenous βarr2 (rARR). Insets show higher magnifications of representative areas. Scale bar represents 5µm. (D) Centrosome-associated fluorescence intensity corresponding to rARR staining in 2KO and 1/2KO MEFs was normalized to that found for WT MEFs. Values are the means (+/− SD) of at least 20 cells from three independent experiments (**: p<0.001). The data show that the signals observed with the rARR antibody in both western-blot and immunofluorescence experiments do depend on the expression of βarr2.
(9.11 MB TIF)

Figure S4 Colocalization of endogenous βarr2 with centrosomal markers. HeLa cells were fixed and stained for both the centrosome, using either mouse monoclonal antibody against γ-tubulin (A and B, green) or rabbit polyclonal antibody against pericentrin (C, green), and βarr2, using either rARR (A, B, red) or gBARR2 (C, red) polyclonal antibodies. Insets show higher magnifications of representative areas. In coloured images, βarr2 staining is in red, centrosome markers in green and nuclei stained with DAPI are in blue. Scale-bars represent 5µm.
(9.23 MB TIF)

Figure S5 Targeting of βarr2 to the centrosome does not depend on microtubules. To confirm that microtubles were effectively affected in live cells treated with nocodazole in Figure 3, control or nocodazole-treated cells were fixed and stained using antibodies against α-tubulin (α-tub) and Giantin, a Golgi marker. As expected, treatment of the cells with nocodazole resulted in disruption of microtubules and dispersion of the Golgi stacks in cell periphery.
(7.08 MB TIF)

Figure S6 βarr2 is neither involved in nucleation nor in anchoring of microtubules to the centrosome. (A) WT or 1/2KO MEFs untreated or treated with nocodazole to depolymerize microtubules were washed, then directly fixed or incubated in DMEM (37°C) for 5 or 10 minutes. Cells were stained for microtubules (α-tubulin, red). Nuclei appear in blue (DAPI). Insets show higher magnifications of microtubule-forming asters around centrosomes. (B) HeLa cells expressing βarr2-GFP tagged fusion were treated with nocodazole then washed in PBS and incubated in pre-warmed DMEM for 5 or 10 minutes. Cells were then fixed and stained for centrosomes (pericentrin) and microtubules (α-tubulin). Insets show higher magnifications of microtubule-forming asters around the centrosome in cells expressing (1) or not βarr2-GFP (2). Scale bars represent 5µm.
(8.79 MB TIF)

Figure S7 Quantification of ciliogenesis in MEF cells using polyglutamylated-tubulin as a marker of PC. (A) WT, 2KO or 1/2KO MEF cells grown on coverslips to confluence and then starved in low serum (0,5%) for 24h, were fixed and stained for polyglutamylated-tubulin (glu-tub). Insets show higher magnifications of PC in each MEF cell lines. In coloured images, polyglutamylated-tubulin staining is in red and nuclei stained with DAPI are in blue. (B) The percentage of primary cilia of MEFs cells is depicted (n>200 cells for each MEF cell lines, from one representative experiment). Scale bars represent 5µm.
(8.50 MB TIF)

Figure S8 Endogenous 14-3-3 and transfected 14-3-3η localized to the centrosome and basal body. (A and B) RPE1 cells grown in high (A) or in low serum (B) conditions to induce ciliogenesis, were fixed and stained for 14-3-3 proteins using a polyclonal antibody recognizing all 14-3-3 isoforms and for either the centrosomal marker γ-tubulin (γ-tub, A) or acetylated-tubulin (AT, B) as indicated. In coloured images, 14-3-3 staining is in red, centrosome and cilia markers in green and nuclei stained with DAPI are in blue. (C) RPE1 cells grown in high serum conditions were transiently transfected with plasmids encoding for a GFP-14-3-3η fusion, fixed and stained for pericentrin (Peric.). In coloured image, 14-3-3 staining is in green, centrosomal markers in red and nuclei stained with DAPI are in blue. (D) RPE1 cells transiently transfected with plasmids encoding for a GFP-14-3-3η fusion were grown for 24 hours in low serum, then fixed and stained for pericentrin (Peric.) and acetylated tubulin (AT). In coloured image, 14-3-3 staining is in green, pericentrin in blue and AT in red. Insets show higher magnifications of representative areas containing the centrosome or the PC. Scale bars represent 5µm.
(8.75 MB TIF)

Figure S9 Ki-67 positive ciliated cells in the absence of βarrs. (A) WT, 1KO or 2KO MEF cells were grown on coverslips to confluence, starved in low serum (0,5%) for 48h, fixed and stained for the Ki-67 proliferation marker and acetylated tubulin (AT). In coloured images, AT staining is in green, Ki-67 in red and nuclei stained with DAPI are in blue. Arrows stress ciliated cells positive for Ki-67. (B) The percentage of Ki-67-negative (quiescent) ciliated cells was calculated (n>200 cells per condition). One representative experiment out of two is shown.
(7.63 MB TIF)

Figure S10 RPE1 cells depleted for βarr2 show ciliogenesis defects. RPE1 cells were treated with control luciferase siRNA (Luc), si-βarr2#2 to deplete βarr2 or si-Kif3A. (A) Expression of Kif3A was analyzed by western-blot. Expression of the γ tubulin (γ-tub) was tested as a control. (B) Cells from the same experiment were also seeded on coverslips and the percentage of cells with primary cilia was determined following AT staining as indicated in Figure 5. Values are the means (+/− SD) of ∼300 cells from a representative experiment done in triplicate.
(8.86 MB TIF)

Figure S11 SST3R does not require βarr2 to be targeted to the PC. (A) WT, 1KO, 2KO or 1/2KO MEF cells were grown on coverslips to confluence, transfected with Flag tagged somatostatin type 3 receptor, starved in low serum (0,5%) for 24h, fixed and stained for Flag tag (red) and acetylated tubulin (AT, green). Nuclei stained with DAPI are in blue. Insets show higher magnifications of representative PCs. Scale bars represent 5µm.
(9.80 MB TIF)

## References

[1] Praetorius HA, Spring KR (2005). A physiological view of the primary cilium.. Annu Rev Physiol.

[2] Michaud EJ, Yoder BK (2006). The primary cilium in cell signaling and cancer.. Cancer Res.

[3] Christensen ST, Pedersen LB, Schneider L, Satir P (2007). Sensory cilia and integration of signal transduction in human health and disease.. Traffic.

[4] Scholey JM (2008). Intraflagellar transport motors in cilia: moving along the cell's antenna.. J Cell Biol.

[5] Fan S, Hurd TW, Liu CJ, Straight SW, Weimbs T (2004). Polarity proteins control ciliogenesis via kinesin motor interactions.. Curr Biol.

[6] Sfakianos J, Togawa A, Maday S, Hull M, Pypaert M (2007). Par3 functions in the biogenesis of the primary cilium in polarized epithelial cells.. J Cell Biol.

[7] Hildebrandt F, Otto E (2005). Cilia and centrosomes: a unifying pathogenic concept for cystic kidney disease?. Nat Rev Genet.

[8] Handel M, Schulz S, Stanarius A, Schreff M, Erdtmann-Vourliotis M (1999). Selective targeting of somatostatin receptor 3 to neuronal cilia.. Neuroscience.

[9] Eggenschwiler JT, Anderson KV (2007). Cilia and developmental signaling.. Annu Rev Cell Dev Biol.

[10] Corbit KC, Aanstad P, Singla V, Norman AR, Stainier DY (2005). Vertebrate Smoothened functions at the primary cilium.. Nature.

[11] May SR, Ashique AM, Karlen M, Wang B, Shen Y (2005). Loss of the retrograde motor for IFT disrupts localization of Smo to cilia and prevents the expression of both activator and repressor functions of Gli.. Dev Biol.

[12] DeWire SM, Ahn S, Lefkowitz RJ, Shenoy SK (2007). Beta-arrestins and cell signaling.. Annu Rev Physiol.

[13] Moore CA, Milano SK, Benovic JL (2007). Regulation of receptor trafficking by GRKs and arrestins.. Annu Rev Physiol.

[14] Kohout TA, Lin FS, Perry SJ, Conner DA, Lefkowitz RJ (2001). beta-Arrestin 1 and 2 differentially regulate heptahelical receptor signaling and trafficking.. Proc Natl Acad Sci U S A.

[15] Benmerah A, Scott M, Poupon V, Marullo S (2003). Nuclear functions for plasma membrane-associated proteins?. Traffic.

[16] Ma L, Pei G (2007). Beta-arrestin signaling and regulation of transcription.. J Cell Sci.

[17] Dawson TM, Arriza JL, Jaworsky DE, Borisy FF, Attramadal H (1993). Beta-adrenergic receptor kinase-2 and beta-arrestin-2 as mediators of odorant-induced desensitization.. Science.

[18] Menco BP (2005). The fine-structural distribution of G-protein receptor kinase 3, beta-arrestin-2, Ca2+/calmodulin-dependent protein kinase II and phosphodiesterase PDE1C2, and a Cl(-)-cotransporter in rodent olfactory epithelia.. J Neurocytol.

[19] Shaner NC, Campbell RE, Steinbach PA, Giepmans BN, Palmer AE (2004). Improved monomeric red, orange and yellow fluorescent proteins derived from Discosoma sp. red fluorescent protein.. Nat Biotechnol.

[20] Azimzadeh J, Bornens M (2007). Structure and duplication of the centrosome.. J Cell Sci.

[21] Bornens M (2002). Centrosome composition and microtubule anchoring mechanisms.. Curr Opin Cell Biol.

[22] Yoder BK (2007). Role of primary cilia in the pathogenesis of polycystic kidney disease.. J Am Soc Nephrol.

[23] Pazour GJ, Dickert BL, Vucica Y, Seeley ES, Rosenbaum JL (2000). Chlamydomonas IFT88 and its mouse homologue, polycystic kidney disease gene tg737, are required for assembly of cilia and flagella.. J Cell Biol.

[24] Yoder BK, Tousson A, Millican L, Wu JH, Bugg CE (2002). Polaris, a protein disrupted in orpk mutant mice, is required for assembly of renal cilium.. Am J Physiol Renal Physiol.

[25] Lin F, Hiesberger T, Cordes K, Sinclair AM, Goldstein LS (2003). Kidney-specific inactivation of the KIF3A subunit of kinesin-II inhibits renal ciliogenesis and produces polycystic kidney disease.. Proc Natl Acad Sci U S A.

[26] Robert A, Margall-Ducos G, Guidotti JE, Bregerie O, Celati C (2007). The intraflagellar transport component IFT88/polaris is a centrosomal protein regulating G1-S transition in non-ciliated cells.. J Cell Sci.

[27] Pan J, Snell W (2007). The primary cilium: keeper of the key to cell division.. Cell.

[28] Gerdes J, Lemke H, Baisch H, Wacker HH, Schwab U (1984). Cell cycle analysis of a cell proliferation-associated human nuclear antigen defined by the monoclonal antibody Ki-67.. J Immunol.

[29] Bridges D, Moorhead GB (2005). 14-3-3 proteins: a number of functions for a numbered protein.. Sci STKE.

[30] Hurd TW, Fan S, Liu CJ, Kweon HK, Hakansson K (2003). Phosphorylation-dependent binding of 14-3-3 to the polarity protein Par3 regulates cell polarity in mammalian epithelia.. Curr Biol.

[31] Xiao K, McClatchy DB, Shukla AK, Zhao Y, Chen M (2007). Functional specialization of beta-arrestin interactions revealed by proteomic analysis.. Proc Natl Acad Sci U S A.

[32] Takeda S, Yonekawa Y, Tanaka Y, Okada Y, Nonaka S (1999). Left-right asymmetry and kinesin superfamily protein KIF3A: new insights in determination of laterality and mesoderm induction by kif3A-/- mice analysis.. J Cell Biol.

[33] Follit JA, Tuft RA, Fogarty KE, Pazour GJ (2006). The intraflagellar transport protein IFT20 is associated with the Golgi complex and is required for cilia assembly.. Mol Biol Cell.

[34] Doxsey S, Zimmerman W, Mikule K (2005). Centrosome control of the cell cycle.. Trends Cell Biol.

[35] Srsen V, Gnadt N, Dammermann A, Merdes A (2006). Inhibition of centrosome protein assembly leads to p53-dependent exit from the cell cycle.. J Cell Biol.

[36] Mikule K, Delaval B, Kaldis P, Jurcyzk A, Hergert P (2007). Loss of centrosome integrity induces p38-p53-p21-dependent G1-S arrest.. Nat Cell Biol.

[37] Girnita L, Shenoy SK, Sehat B, Vasilcanu R, Vasilcanu D (2007). Beta-arrestin and Mdm2 mediate IGF-1 receptor-stimulated ERK activation and cell cycle progression.. J Biol Chem.

[38] Kang J, Shi Y, Xiang B, Qu B, Su W (2005). A nuclear function of beta-arrestin1 in GPCR signaling: regulation of histone acetylation and gene transcription.. Cell.

[39] Piu F, Gauthier NK, Wang F (2006). Beta-arrestin 2 modulates the activity of nuclear receptor RAR beta2 through activation of ERK2 kinase.. Oncogene.

[40] Shenoy SK, McDonald PH, Kohout TA, Lefkowitz RJ (2001). Regulation of receptor fate by ubiquitination of activated beta 2-adrenergic receptor and beta-arrestin.. Science.

[41] Wang P, Gao H, Ni Y, Wang B, Wu Y (2003). Beta-arrestin 2 functions as a G-protein-coupled receptor-activated regulator of oncoprotein Mdm2.. J Biol Chem.

[42] Boularan C, Scott MG, Bourougaa K, Bellal M, Esteve E (2007). beta-arrestin 2 oligomerization controls the Mdm2-dependent inhibition of p53.. Proc Natl Acad Sci U S A.

[43] Qin H, Wang Z, Diener D, Rosenbaum J (2007). Intraflagellar transport protein 27 is a small G protein involved in cell-cycle control.. Curr Biol.

[44] Kovacs JJ, Whalen EJ, Liu R, Xiao K, Kim J (2008). {beta}-Arrestin-Mediated Localization of Smoothened to the Primary Cilium.. Science.

[45] Wheatley DN (1969). Cilia in cell-cultured fibroblasts. I. On their occurrence and relative frequencies in primary cultures and established cell lines.. J Anat.

[46] Frew IJ, Thoma CR, Georgiev S, Minola A, Hitz M (2008). pVHL and PTEN tumour suppressor proteins cooperatively suppress kidney cyst formation.. Embo J.

[47] Spektor A, Tsang WY, Khoo D, Dynlacht BD (2007). Cep97 and CP110 suppress a cilia assembly program.. Cell.

[48] Calvert PD, Strissel KJ, Schiesser WE, Pugh EN, Arshavsky VY (2006). Light-driven translocation of signaling proteins in vertebrate photoreceptors.. Trends Cell Biol.

[49] Marszalek JR, Liu X, Roberts EA, Chui D, Marth JD (2000). Genetic evidence for selective transport of opsin and arrestin by kinesin-II in mammalian photoreceptors.. Cell.

[50] van Veen T, Elofsson R, Hartwig HG, Gery I, Mochizuki M (1986). Retinal S-antigen: immunocytochemical and immunochemical studies on distribution in animal photoreceptors and pineal organs.. Exp Biol.

[51] Reid DM, Loeffler KU, Campbell AM, Forrester JV (1987). Electron immunocytochemical localization of retinal S-antigen with a rat monoclonal antibody.. Exp Eye Res.

[52] McGinnis JF, Matsumoto B, Whelan JP, Cao W (2002). Cytoskeleton participation in subcellular trafficking of signal transduction proteins in rod photoreceptor cells.. J Neurosci Res.

[53] Peterson JJ, Tam BM, Moritz OL, Shelamer CL, Dugger DR (2003). Arrestin migrates in photoreceptors in response to light: a study of arrestin localization using an arrestin-GFP fusion protein in transgenic frogs.. Exp Eye Res.

[54] Corbit KC, Shyer AE, Dowdle WE, Gaulden J, Singla V (2008). Kif3a constrains beta-catenin-dependent Wnt signalling through dual ciliary and non-ciliary mechanisms.. Nat Cell Biol.

[55] Kovacs JJ, Whalen EJ, Liu R, Xiao K, Kim J (2008). Beta-arrestin-mediated localization of smoothened to the primary cilium.. Science.

[56] Berbari NF, Lewis JS, Bishop GA, Askwith CC, Mykytyn K (2008). Bardet-Biedl syndrome proteins are required for the localization of G protein-coupled receptors to primary cilia.. Proc Natl Acad Sci U S A.

[57] Palmitessa A, Hess HA, Bany IA, Kim YM, Koelle MR (2005). Caenorhabditus elegans arrestin regulates neural G protein signaling and olfactory adaptation and recovery.. J Biol Chem.

[58] Ge H, Krishnan P, Liu L, Krishnan B, Davis RL (2006). A Drosophila nonvisual arrestin is required for the maintenance of olfactory sensitivity.. Chem Senses.

[59] Scott MG, Le Rouzic E, Perianin A, Pierotti V, Enslen H (2002). Differential nucleocytoplasmic shuttling of beta-arrestins. Characterization of a leucine-rich nuclear export signal in beta-arrestin2.. J Biol Chem.

[60] Taipale J, Chen JK, Cooper MK, Wang B, Mann RK (2000). Effects of oncogenic mutations in Smoothened and Patched can be reversed by cyclopamine.. Nature.

[61] Piel M, Meyer P, Khodjakov A, Rieder CL, Bornens M (2000). The respective contributions of the mother and daughter centrioles to centrosome activity and behavior in vertebrate cells.. J Cell Biol.

[62] Wolff A, de Nechaud B, Chillet D, Mazarguil H, Desbruyeres E (1992). Distribution of glutamylated alpha and beta-tubulin in mouse tissues using a specific monoclonal antibody, GT335.. Eur J Cell Biol.

[63] Ahn S, Nelson CD, Garrison TR, Miller WE, Lefkowitz RJ (2003). Desensitization, internalization, and signaling functions of beta-arrestins demonstrated by RNA interference.. Proc Natl Acad Sci U S A.

[64] Gesty-Palmer D, Chen M, Reiter E, Ahn S, Nelson CD (2006). Distinct beta-arrestin- and G protein-dependent pathways for parathyroid hormone receptor-stimulated ERK1/2 activation.. J Biol Chem.

[65] Borck G, Molla-Herman A, Boddaert N, Encha-Razavi F, Philippe A (2008). Clinical, cellular, and neuropathological consequences of AP1S2 mutations: further delineation of a recognizable X-linked mental retardation syndrome.. Hum Mutat.

[66] Burtey A, Schmid EM, Ford MG, Rappoport JZ, Scott MG (2007). The conserved isoleucine-valine-phenylalanine motif couples activation state and endocytic functions of beta-arrestins.. Traffic.

