# Supplementary figures and images for: Targeting of β-Arrestin2 to the Centrosome and Primary Cilium: Role in Cell Proliferation Control
Source: PLoS One. 2008 Nov 14;3(11):e3728. doi: 10.1371/journal.pone.0003728 (PMC2579577; doi:10.1371/journal.pone.0003728)

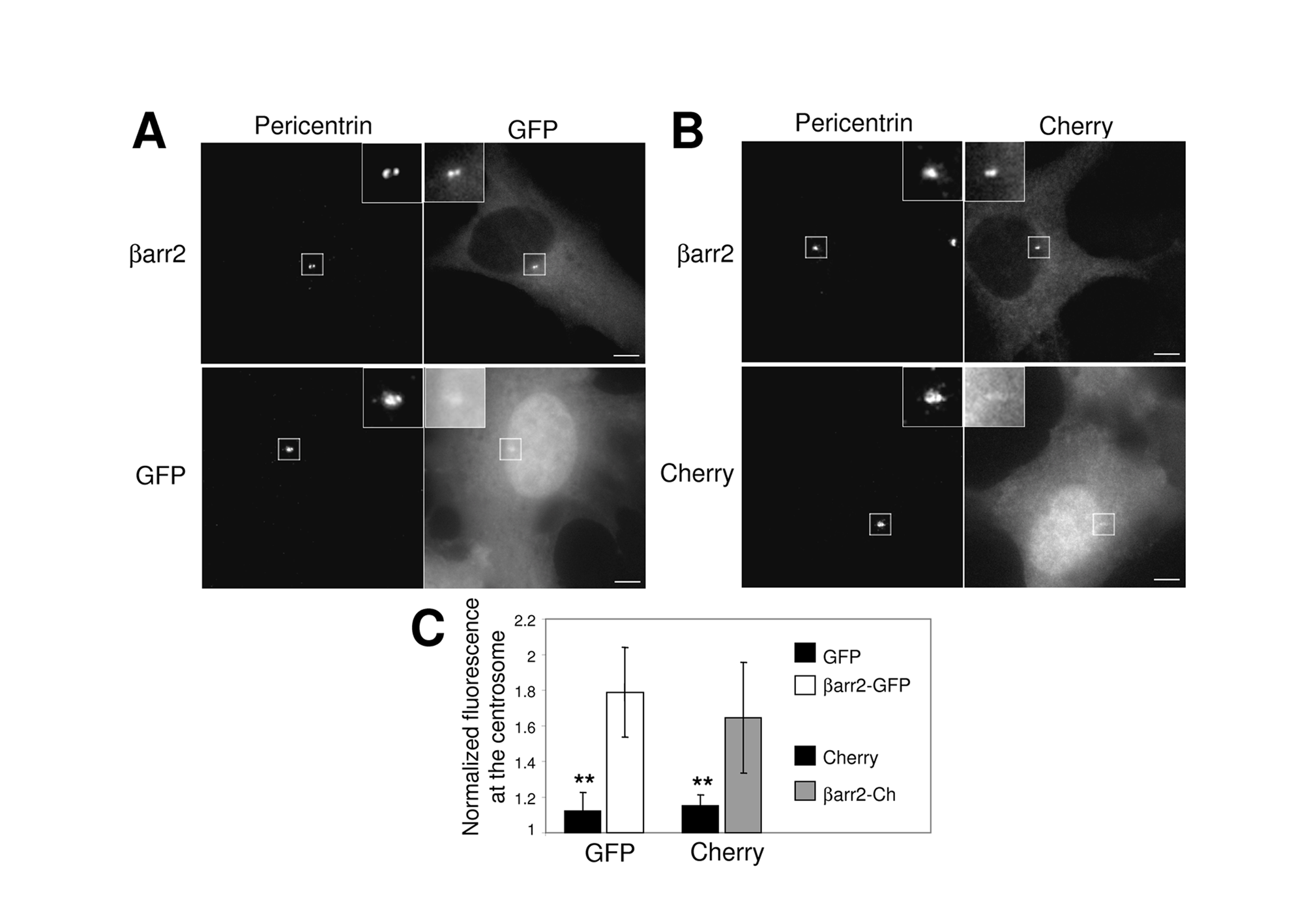

Supplement: Figure S1 — Targeting of GFP and Cherry βarr2 fusion at the centrosome in RPE1 cells. RPE1 (retinal pigment epithelial) cells were transiently transfected with plasmids encoding for βarr2-GFP fusion or GFP alone (A), or βarr2-Cherry fusion or Cherry alone (B), then fixed and stained for the centrosomal marker pericentrin. Insets show higher magnifications of representative areas. Scale bar represents 5µm. (C) Pericentrin staining was used to determine the centrosome-associated fluorescence intensity for GFP or Cherry (see Methods) which was then normalized to the cytoplasmic signal in the same cells. Values are the means (+/− SD) of at least 15 cells from three independent experiments ( **: p<0.001). (8.56 MB TIF) [file pone.0003728.s001.tif]

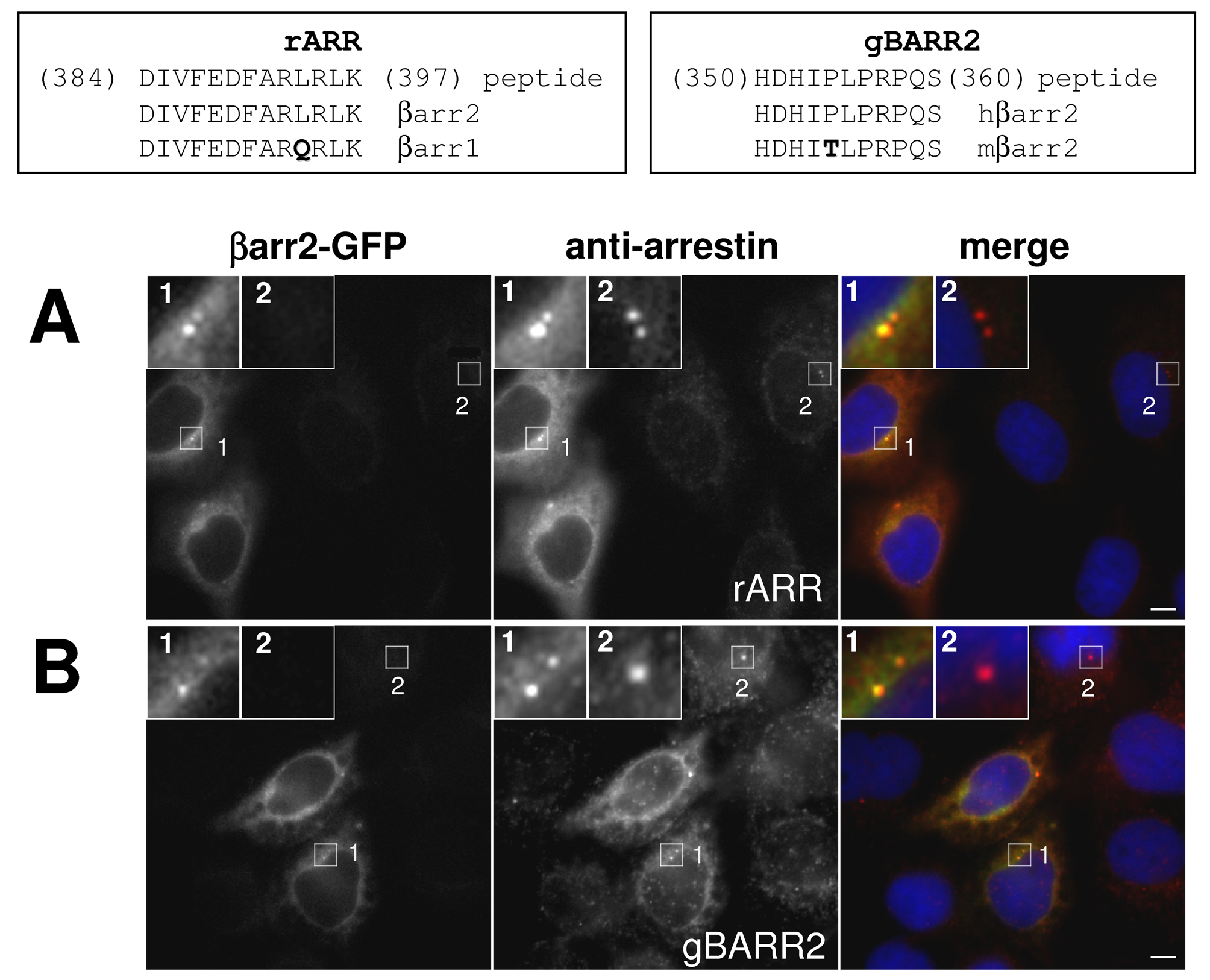

Supplement: Figure S2 — Characterization of anti-βarr2 antibodies. Description of the immunogenic peptides used to generate anti-βarr2 polyclonal antibodies: The rARR rabbit polyclonal antibody is sold as an antibody against both βarr2 and βarr1 but a single amino-acid difference in the immunogenic peptide makes it more specific for βarr2. The gBARR2 goat polyclonal was raised against a peptide specific of human βarr2 and not conserved in βarr1. However, a single amino acid difference between human and rodent βarr2 is likely to explain its poor reactivity against murine endogenous βarr2 observed in both western blot and immunofluorescence (data not shown). HeLa cells were transiently transfected with plasmids encoding for βarr2-GFP, then fixed and stained for the anti-βarr2 antibodies, including the rabbit polyclonal rARR anti-arrestin (A) and the goat polyclonal gBARR2 anti βarrestin2 (B). In coloured images, βarr2-GFP staining is in green, endogenous βarr2 in red and nuclei stained with DAPI are in blue. Insets show higher magnifications of representative areas corresponding to the centrosome containing region of cells expressing (1) or not (2) GFP-βarr2 in the same field. GFP-βarr2 expressing cells showed increase staining with the anti-βarr2 antibodies showing that they did work for immunofluorescence. In non-transfected cells, the antibodies showed a diffuse staining in the cytoplasm and illuminated two bright spots, suggesting that both antibodies are able to detect both overexpressed and endogenous βarr2. Scale bars represent 5µm. (9.82 MB TIF) [file pone.0003728.s002.tif]

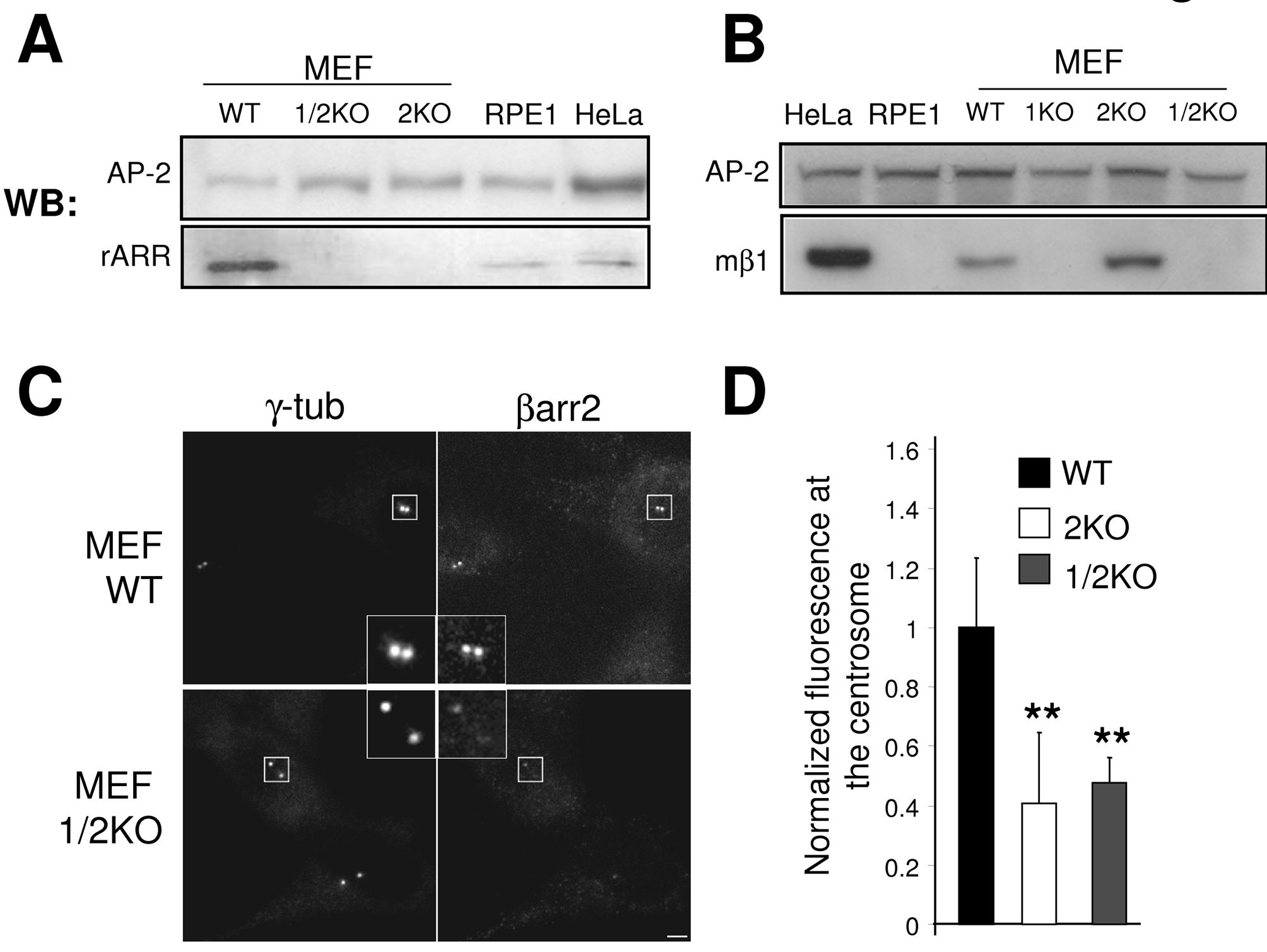

Supplement: Figure S3 — The rARR antibody is specific for βarr2 and stains the centrosome. It has to be stressed here that, independently of the commercial source, we observed a variability between batches of commercial anti-βarr2 antibodies; while almost all batches did detect overexpressed βarr2, some were unable to detect endogenous βarr2 in neither western-blot or immunofluorescence experiments. The efficiency of each batch was then tested by western-blot using WT and βarrs-KO MEFs as described below. (A and B) βarr2 expression was assessed in mouse embryonic fibroblasts (MEFs) derived from wild type (WT), βarr2 deficient (2KO), both βarr1 and βarr2 deficient (1/2KO) mice, RPE1 (retinal pigment epithelial cells) or HeLa cells by western blotting (WB) using the rabbit polyclonal antibody against βarr2 (rARR, (A)) or a monoclonal antibody against βarr1 (mβ1, (B)). An antibody against α-adaptin subunit of the clathrin adaptor complex AP2 was used as a loading control. (C) WT or 1/2KO MEFs were fixed and stained for the centrosomal marker γ-tubulin (γ-tub) and endogenous βarr2 (rARR). Insets show higher magnifications of representative areas. Scale bar represents 5µm. (D) Centrosome-associated fluorescence intensity corresponding to rARR staining in 2KO and 1/2KO MEFs was normalized to that found for WT MEFs. Values are the means (+/− SD) of at least 20 cells from three independent experiments (**: p<0.001). The data show that the signals observed with the rARR antibody in both western-blot and immunofluorescence experiments do depend on the expression of βarr2. (9.11 MB TIF) [file pone.0003728.s003.tif]

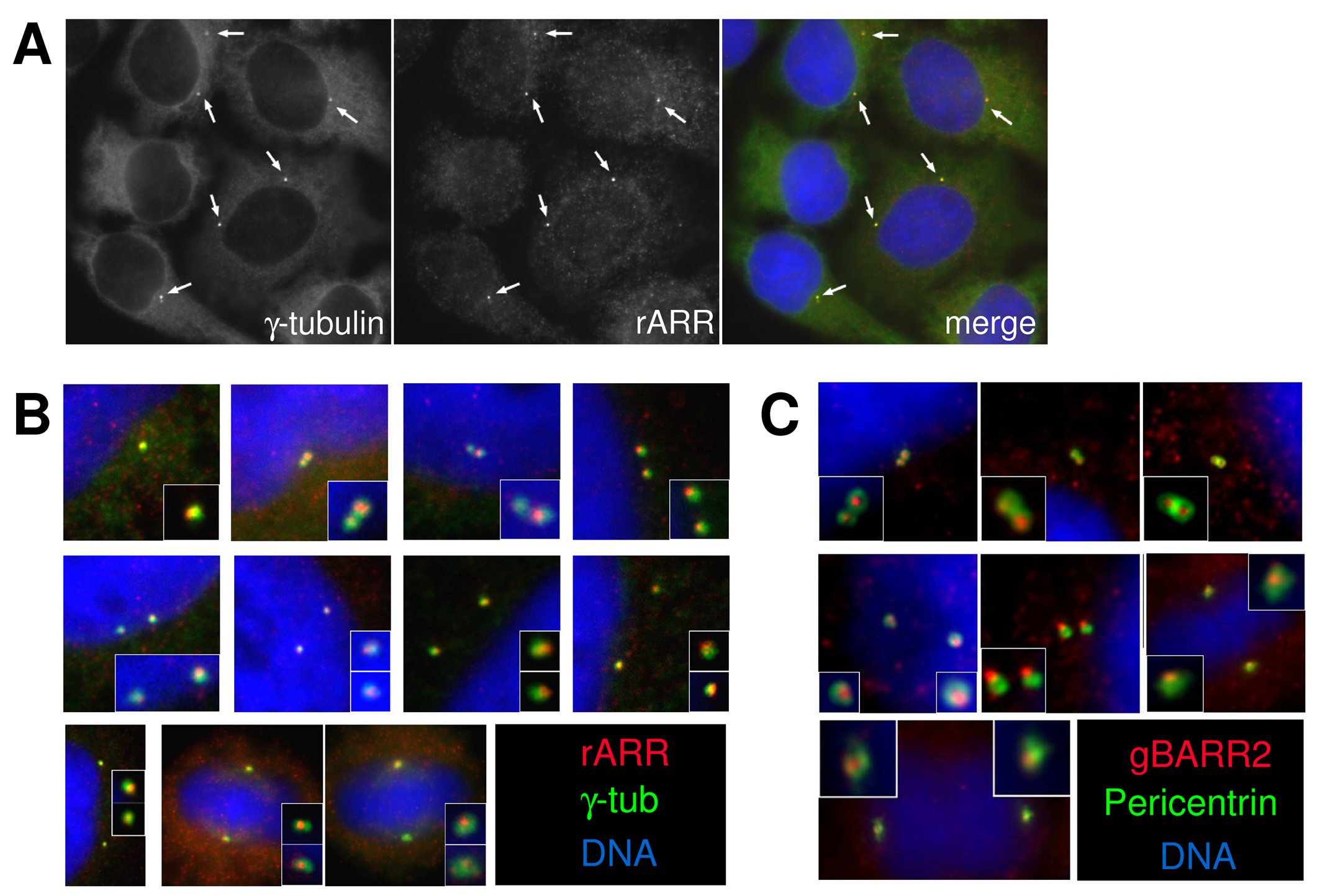

Supplement: Figure S4 — Colocalization of endogenous βarr2 with centrosomal markers. HeLa cells were fixed and stained for both the centrosome, using either mouse monoclonal antibody against γ-tubulin (A and B, green) or rabbit polyclonal antibody against pericentrin (C, green), and βarr2, using either rARR (A, B, red) or gBARR2 (C, red) polyclonal antibodies. Insets show higher magnifications of representative areas. In coloured images, βarr2 staining is in red, centrosome markers in green and nuclei stained with DAPI are in blue. Scale-bars represent 5µm. (9.23 MB TIF) [file pone.0003728.s004.tif]

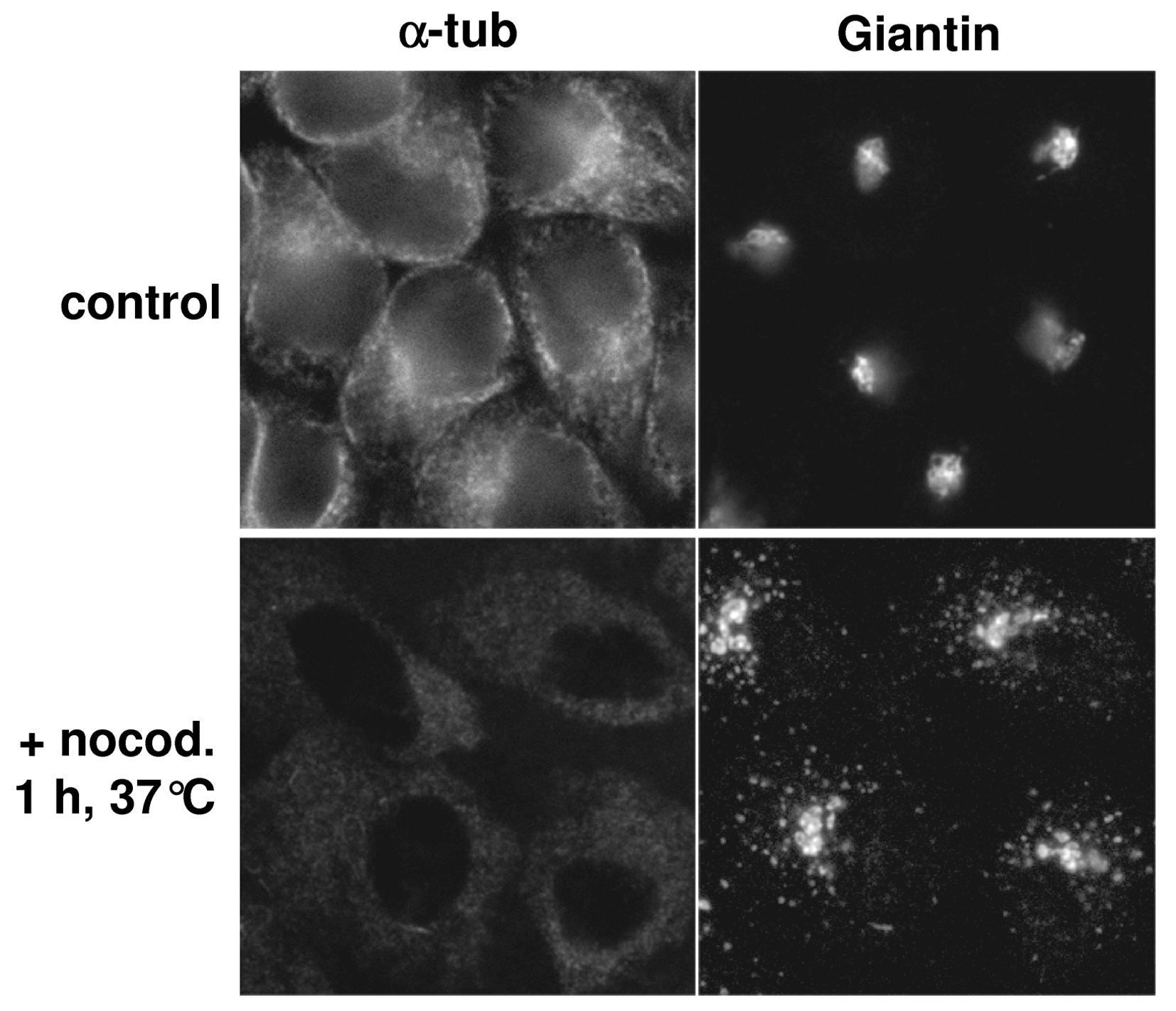

Supplement: Figure S5 — Targeting of βarr2 to the centrosome does not depend on microtubules. To confirm that microtubles were effectively affected in live cells treated with nocodazole in Figure 3, control or nocodazole-treated cells were fixed and stained using antibodies against α-tubulin (α-tub) and Giantin, a Golgi marker. As expected, treatment of the cells with nocodazole resulted in disruption of microtubules and dispersion of the Golgi stacks in cell periphery. (7.08 MB TIF) [file pone.0003728.s005.tif]

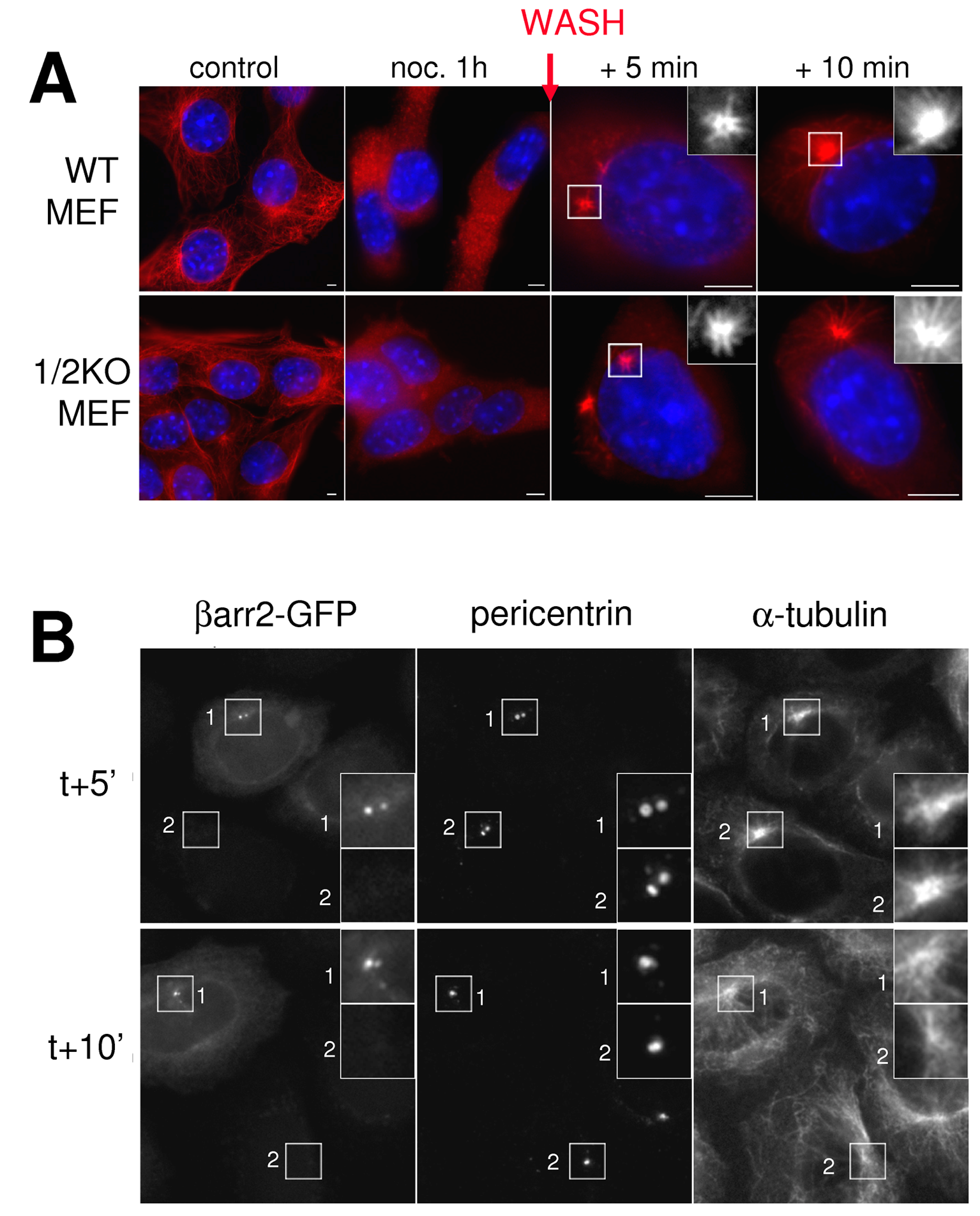

Supplement: Figure S6 — βarr2 is neither involved in nucleation nor in anchoring of microtubules to the centrosome. (A) WT or 1/2KO MEFs untreated or treated with nocodazole to depolymerize microtubules were washed, then directly fixed or incubated in DMEM (37°C) for 5 or 10 minutes. Cells were stained for microtubules (α-tubulin, red). Nuclei appear in blue (DAPI). Insets show higher magnifications of microtubule-forming asters around centrosomes. (B) HeLa cells expressing βarr2-GFP tagged fusion were treated with nocodazole then washed in PBS and incubated in pre-warmed DMEM for 5 or 10 minutes. Cells were then fixed and stained for centrosomes (pericentrin) and microtubules (α-tubulin). Insets show higher magnifications of microtubule-forming asters around the centrosome in cells expressing (1) or not βarr2-GFP (2). Scale bars represent 5µm. (8.79 MB TIF) [file pone.0003728.s006.tif]

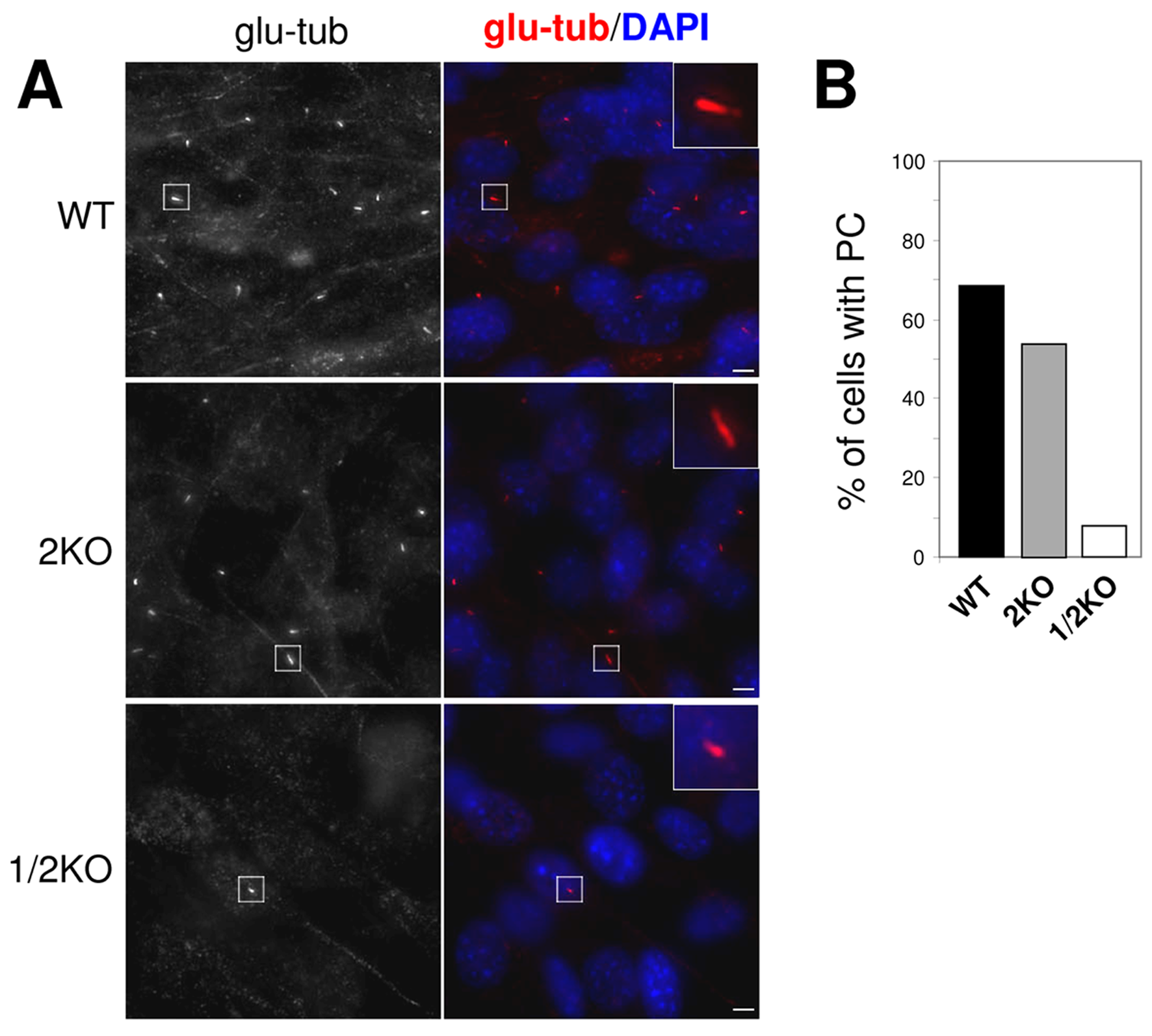

Supplement: Figure S7 — Quantification of ciliogenesis in MEF cells using polyglutamylated-tubulin as a marker of PC. (A) WT, 2KO or 1/2KO MEF cells grown on coverslips to confluence and then starved in low serum (0,5%) for 24h, were fixed and stained for polyglutamylated-tubulin (glu-tub). Insets show higher magnifications of PC in each MEF cell lines. In coloured images, polyglutamylated-tubulin staining is in red and nuclei stained with DAPI are in blue. (B) The percentage of primary cilia of MEFs cells is depicted (n>200 cells for each MEF cell lines, from one representative experiment). Scale bars represent 5µm. (8.50 MB TIF) [file pone.0003728.s007.tif]

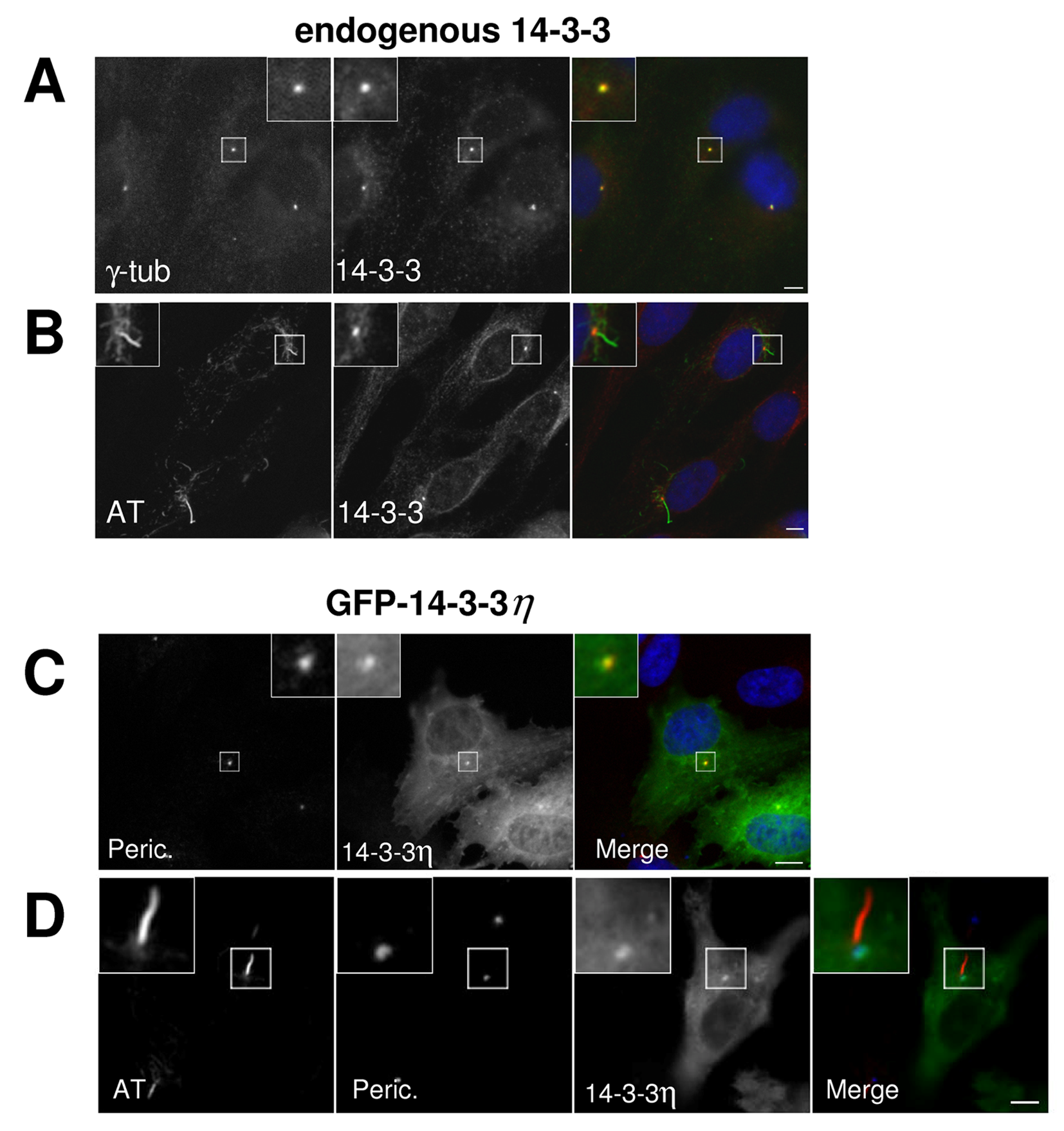

Supplement: Figure S8 — Endogenous 14-3-3 and transfected 14-3-3η localized to the centrosome and basal body. (A and B) RPE1 cells grown in high (A) or in low serum (B) conditions to induce ciliogenesis, were fixed and stained for 14-3-3 proteins using a polyclonal antibody recognizing all 14-3-3 isoforms and for either the centrosomal marker γ-tubulin (γ-tub, A) or acetylated-tubulin (AT, B) as indicated. In coloured images, 14-3-3 staining is in red, centrosome and cilia markers in green and nuclei stained with DAPI are in blue. (C) RPE1 cells grown in high serum conditions were transiently transfected with plasmids encoding for a GFP-14-3-3η fusion, fixed and stained for pericentrin (Peric.). In coloured image, 14-3-3 staining is in green, centrosomal markers in red and nuclei stained with DAPI are in blue. (D) RPE1 cells transiently transfected with plasmids encoding for a GFP-14-3-3η fusion were grown for 24 hours in low serum, then fixed and stained for pericentrin (Peric.) and acetylated tubulin (AT). In coloured image, 14-3-3 staining is in green, pericentrin in blue and AT in red. Insets show higher magnifications of representative areas containing the centrosome or the PC. Scale bars represent 5µm. (8.75 MB TIF) [file pone.0003728.s008.tif]

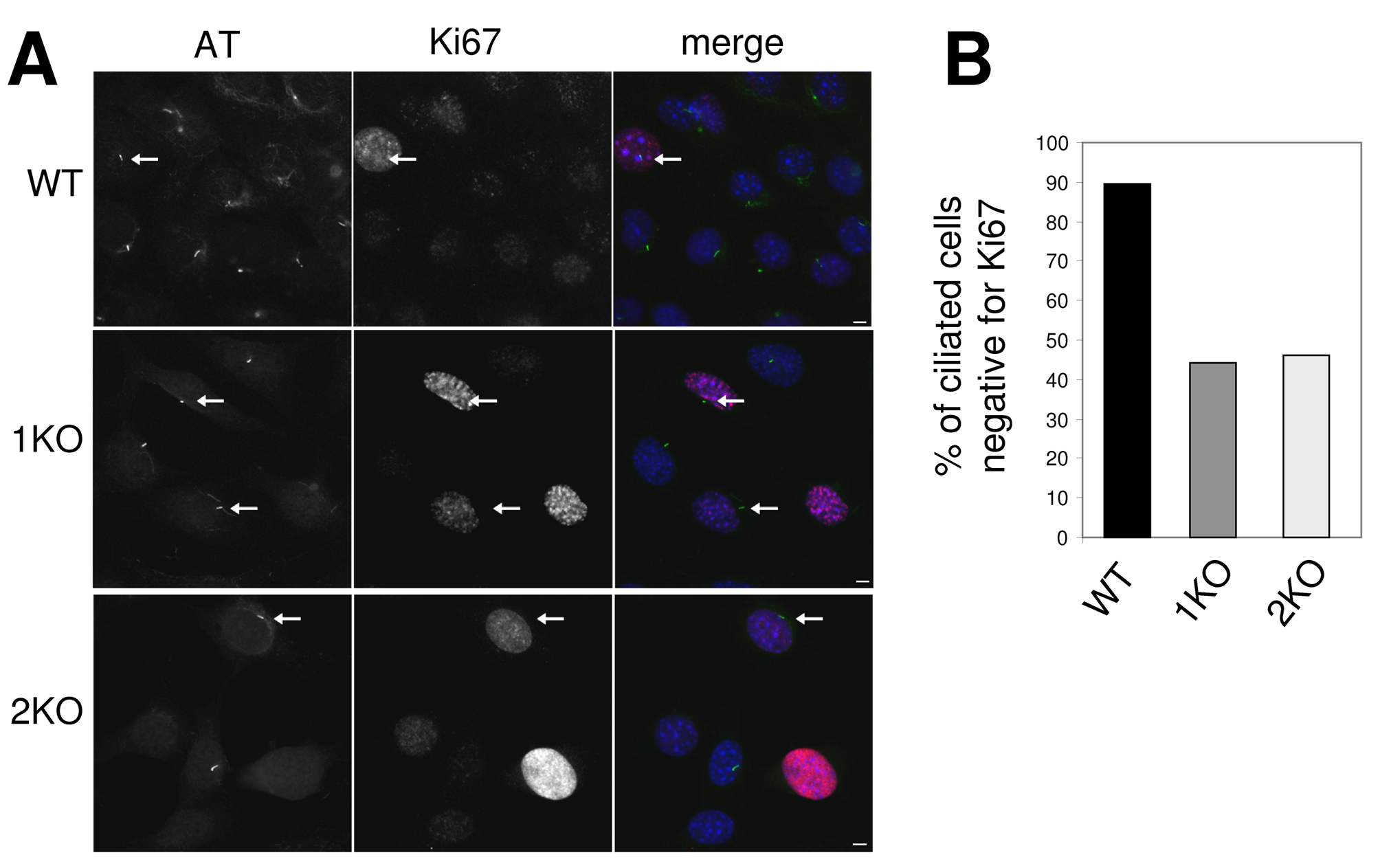

Supplement: Figure S9 — Ki-67 positive ciliated cells in the absence of βarrs. (A) WT, 1KO or 2KO MEF cells were grown on coverslips to confluence, starved in low serum (0,5%) for 48h, fixed and stained for the Ki-67 proliferation marker and acetylated tubulin (AT). In coloured images, AT staining is in green, Ki-67 in red and nuclei stained with DAPI are in blue. Arrows stress ciliated cells positive for Ki-67. (B) The percentage of Ki-67-negative (quiescent) ciliated cells was calculated (n>200 cells per condition). One representative experiment out of two is shown. (7.63 MB TIF) [file pone.0003728.s009.tif]

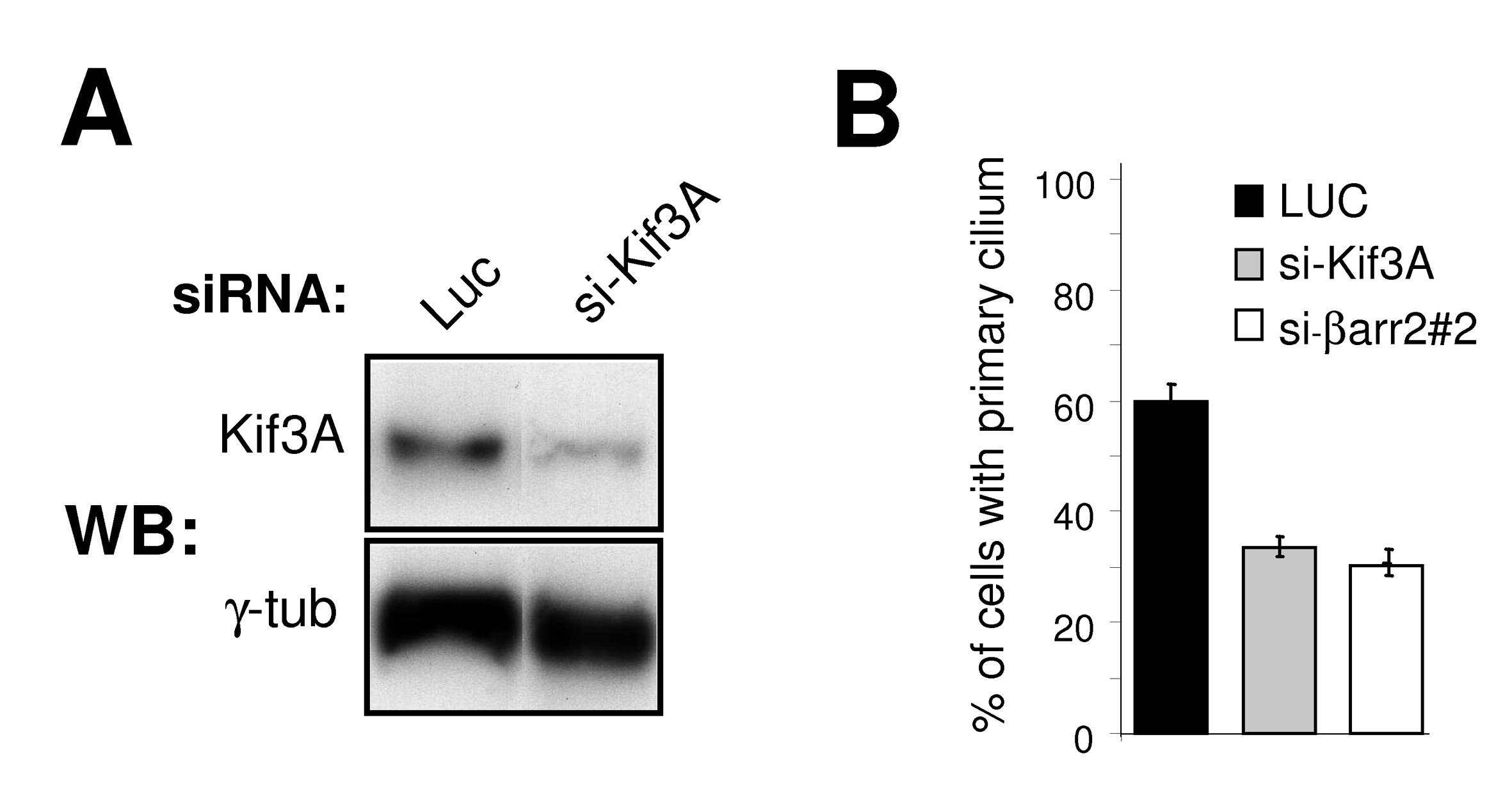

Supplement: Figure S10 — RPE1 cells depleted for βarr2 show ciliogenesis defects. RPE1 cells were treated with control luciferase siRNA (Luc), si-βarr2#2 to deplete βarr2 or si-Kif3A. (A) Expression of Kif3A was analyzed by western-blot. Expression of the γ tubulin (γ-tub) was tested as a control. (B) Cells from the same experiment were also seeded on coverslips and the percentage of cells with primary cilia was determined following AT staining as indicated in Figure 5. Values are the means (+/− SD) of ∼300 cells from a representative experiment done in triplicate. (8.86 MB TIF) [file pone.0003728.s010.tif]

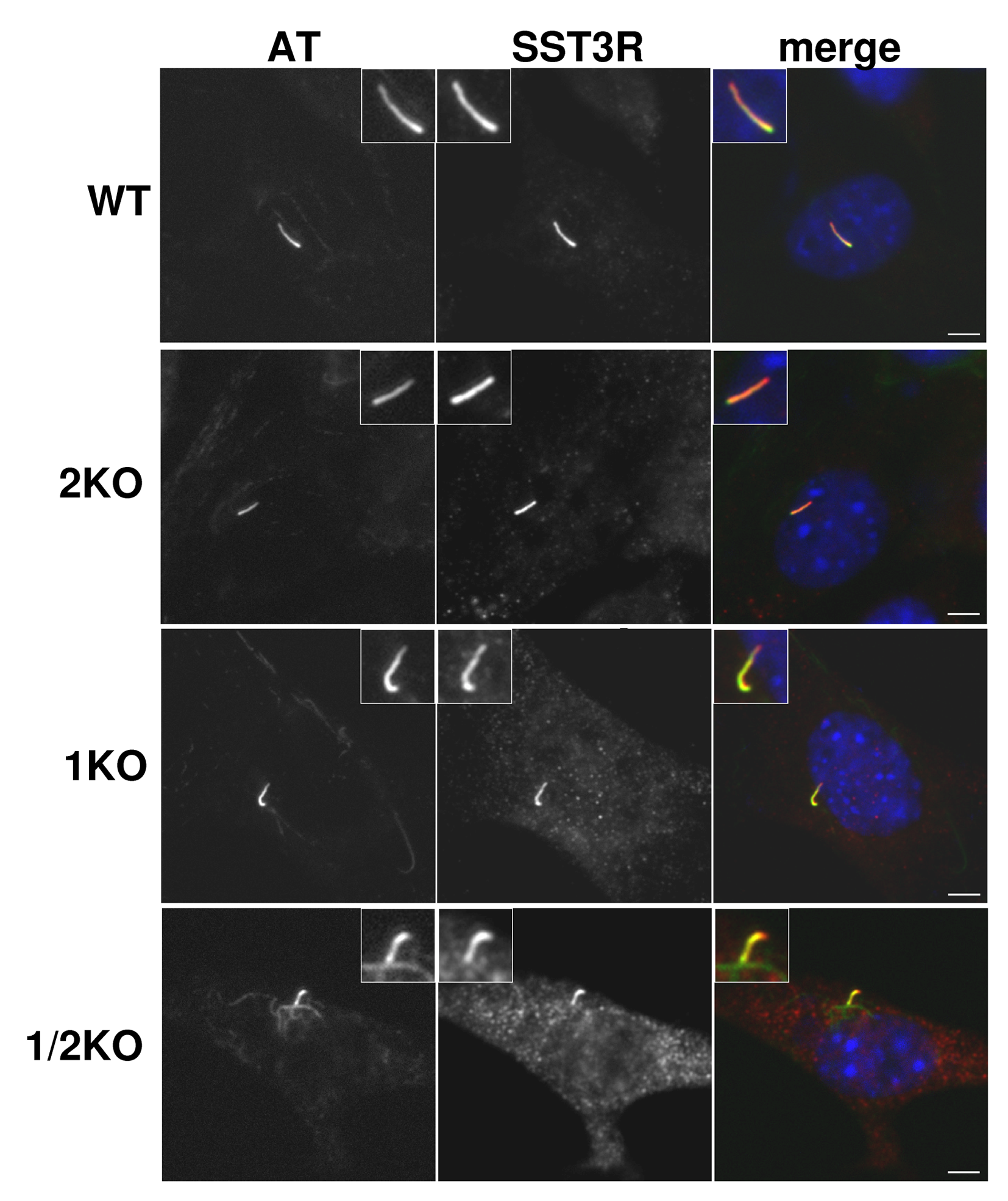

Supplement: Figure S11 — SST3R does not require βarr2 to be targeted to the PC. (A) WT, 1KO, 2KO or 1/2KO MEF cells were grown on coverslips to confluence, transfected with Flag tagged somatostatin type 3 receptor, starved in low serum (0,5%) for 24h, fixed and stained for Flag tag (red) and acetylated tubulin (AT, green). Nuclei stained with DAPI are in blue. Insets show higher magnifications of representative PCs. Scale bars represent 5µm. (9.80 MB TIF) [file pone.0003728.s011.tif]
